# A fluorescein-conjugated somatostatin receptor antagonist adapter for chimeric antigen receptor T cell therapy of meningioma

**DOI:** 10.1093/noajnl/vdag186

**Published:** 2026-07-21

**Authors:** Jiawei Chen, Christian Pellegrino, Maximilian Mastall, Frank Szulzewsky, Mitchell Bijnen, Hannah J Van Hove, Nicholas Favalli, Gabriele Bassi, Vlad Moisoiu, Flavio Vasella, Luis Padevit, Marian C Neidert, Allison Shelbourn, Patrick J Cimino, Regina Reimann, Patrick Roth, Markus G Manz, Melanie Greter, Eric C Holland, Dario Neri, Michael Weller, Hans-Georg Wirsching

**Affiliations:** Department of Neurology, University Hospital and University of Zurich, Zurich, Switzerland; Department of Neurosurgery, Huashan Hospital, Shanghai Medical College, Fudan University, Shanghai, China; Department of Medical Oncology and Hematology, University Hospital and University of Zurich, Zurich, Switzerland; Department of Neurology, University Hospital and University of Zurich, Zurich, Switzerland; Department of Neurosurgery, Clinical Neurosciences Center, University of Utah, Salt Lake City, Utah, USA; Huntsman Cancer Institute, University of Utah Health Sciences Center, Salt Lake City, Utah, USA; Institute of Experimental Immunology, University of Zurich, Zurich, Switzerland; Institute of Experimental Immunology, University of Zurich, Zurich, Switzerland; Philochem AG, Otelfingen, Switzerland; Philochem AG, Otelfingen, Switzerland; Department of Neurology, University Hospital and University of Zurich, Zurich, Switzerland; Department of Neurosurgery, Clinical Neuroscience Center, University Hospital and University of Zurich, Zurich, Switzerland; Department of Neurosurgery, Clinical Neuroscience Center, University Hospital and University of Zurich, Zurich, Switzerland; Department of Neurosurgery, Cantonal Hospital St. Gallen, St.Gallen, Switzerland; Neuropathology Unit, Surgical Neurology Branch, National Institute of Neurological Disorders and Stroke, National Institutes of Health, Bethesda, Maryland, USA; Molecular Neuropathology, Department of Pathology and Laboratory Medicine, Children’s National Research Institute and George Washington University School of Medicine and Health Sciences, Washington, District of Columbia, USA; Institute of Neuropathology, University Hospital and University of Zurich, Zurich, Switzerland; Department of Neurology, University Hospital and University of Zurich, Zurich, Switzerland; Department of Medical Oncology and Hematology, University Hospital and University of Zurich, Zurich, Switzerland; Institute of Experimental Immunology, University of Zurich, Zurich, Switzerland; Human Biology Division, Fred Hutchinson Cancer Center, Seattle, Washington, USA; Philochem AG, Otelfingen, Switzerland; Department of Chemistry and Applied Biosciences, Federal Institute of Technology (ETH), Zurich, Switzerland; Department of Neurology, University Hospital and University of Zurich, Zurich, Switzerland; Department of Neurology, University Hospital and University of Zurich, Zurich, Switzerland; Department of Neurology, Cantonal Hospital Winterthur, Zurich, Switzerland

**Keywords:** brain tumor, cancer immunotherapy, CAR-T cells, genetically engineered meningioma mouse model

## Abstract

**Background:**

A significant proportion of meningiomas are resistant to current treatments. Somatostatin receptor 2 (SSTR2) is highly and consistently expressed in most meningiomas, providing a promising target for localized chimeric antigen receptor (CAR)-T cell therapy. Short-lived small-molecule CAR adapters can potentially prevent CAR-T cell exhaustion in solid tumors by alternating between active and resting states.

**Methods:**

We developed the CAR adapter peptide Octofluo, which combines fluorescein-5-isothiocyanate (FITC) with a high-avidity SSTR2 antagonist. After determining its biodistribution, killing efficiency of CAR-T cells plus Octofluo against human meningioma was evaluated *in vitro* and *ex vivo*. Therapeutic capacity was assessed *in vivo* against xenograft and syngeneic genetically engineered mouse meningioma models.

**Results:**

We herein demonstrate rapid tissue diffusion and transient tumor persistence for only a few hours after intravenous administration of Octofluo, making a suitable switch for on-demand CAR-T cell activation. Nanomolar concentrations of Octofluo effectively directed FITC-specific CAR-T cells against SSTR2-expressing meningioma cells. Combined intratumoral CAR-T cell delivery and intravenous octofluo infusion at periodic intervals showed limited efficacy in an immunocompromised xenograft model but cured most mice with an intact immune system harboring highly aggressive, genetically induced higher grade meningiomas. Cures were accompanied by CAR-T cell expansion and an endogenous T cell response, suggesting a role for the host immune system in tumor elimination. *Ex vivo* lysis of patient-derived meningioma cells was observed.

**Conclusions:**

The combination of systemic octofluo administration and locally applied CAR-T cells is a promising strategy for future clinical development for patients with refractory meningiomas.

Key Points

- Octofluo is a SSTR2-targeted, FITC-linked bispecific CAR-T cell engager.
- Octofluo plus FITC-targeted CAR-T cells are active against SSTR2+ meningioma.
- Octofluo plus FITC-targeted CAR-T cells induce a host anti-tumor immune response.

Importance of the Study

A substantial proportion of meningiomas recur despite bimodal therapy with neurosurgery and radiotherapy. Somatostatin receptor 2 (SSTR2) is highly expressed in most meningiomas and has proven a suitable target for the delivery of therapeutic agents. We therefore synthesized and characterized the pharmacokinetics of a short-lived, bispecific peptide comprised of a high affinity octameric SSTR2 antagonist (octofluo) for linkage of the SSTR2-targeted moiety to fluorescein-5-isothiocyanate (FITC). Systemic administration of this molecule combined with local delivery of FITC-targeted chimeric antigen receptor (CAR-T) cells proved highly effective against several meningioma models, including a genetically engineered murine model of malignant meningioma. The use of a CAR directed against FITC, ie a synthetic target, along with local CAR-T cell delivery limits the risk of off-target toxicity. CAR-T cell exhaustion, one key obstacles of CAR-T cell therapies for solid tumors, may moreover be limited by exploiting the dependence of CAR-T cell activity on adapter availability through metronomic application.

Chimeric antigen receptor (CAR)-T cell therapy has shown great success in the treatment of a subset of hematological malignancies, which led to the clinical approval of several CAR-T cell products.^1^^,^^2^ The key challenges to the clinical translation of CAR-T cell therapies are posed by treatment-associated toxicities on one hand^3-7^ and limited efficacy on the other hand.^8-10^ Strategies to improve the safety and efficacy of CAR-T cell therapies include adapter CAR-T cell (AdCAR-T) systems, in which the AdCAR-T cell is directed against a tag on bispecific adapter molecules.^11-13^ Thereby, the CAR adapter molecule is composed of a tag and an antigen binding moiety, which confers an “on/off” switch for the titration of CAR-T cell activity via its concentration. Employing small organic ligands with a short half-life to bridge CAR-T cells and tumor cells would in theory enable timely regulation and optimization of CAR activity by modulating infusion intervals of the adapter molecule.^14-17^

We have addressed this conceptual advance of CAR-T cell therapies for solid tumors paradigmatically in meningiomas, the most common primary central nervous system tumors in adults.^18^^,^^19^ Most meningiomas can be cured by neurosurgery alone, but association with critical anatomic structures may preclude gross total resection.^20^^,^^21^ Moreover, approximately one in five meningiomas exhibit histopathologic or molecular signs of higher grade^18^^,^^22^^,^^23^ and lower grade meningiomas can likewise recur even decades after a successful initial resection. Repeat neurosurgery and radiotherapy are the only established therapeutic options for recurrent or progressive meningioma,^24^ whereas cytostatics, anti-angiogenics, small molecule inhibitors and other systemic treatment approaches exert limited clinical efficacy at best.^25^ Therefore, there is an urgent medical need for better therapies of irresectable, recurrent or progressive meningioma.

The G protein-coupled somatostatin receptor 2A (SSTR2) is abundantly expressed by the majority of meningiomas.^26^ Positron emission tomography utilizing the SSTR2 agonist DOTATATE has emerged as a diagnostic tool to differentiate tumor from nonneoplastic tissue and to monitor tumor growth of meningiomas.^27-29^ Moreover, DOTATATE linked to Lutethium-177 has been explored as a therapeutic approach to deliver ionizing irradiation specifically to meningiomas.^30^^,^^31^ The feasibility of SSTR2-directed PET tracer and radioligand delivery proved the concept and paved the way for the development of SSTR2-directed therapies.

Here, we report a proof-of-concept study of the fluorescein isothiocyanate (FITC)-conjugated, SSTR2-targeted adapter molecule octofluo combined with CAR-T cells targeting FITC to treat meningioma.

## Methods

### Experimental Model and Subject Details

#### Cell lines

HEK293T, Ben-Men-1, and IOMM-Lee cells were maintained in Dulbecco’s modified Eagle’s medium (DMEM), containing 2mM L-glutamine, 1% penicillin/streptomycin, and 10% fetal calf serum (FCS). Platinum-E was cultured in DMEM with 10% FCS, 2 mmol/L Glutamine, 1% penicillin/streptomycin, 1 μg/ml puromycin, 10 μg/ml blasticidin. DF-1 cells were maintained in DMEM containing 2 mM L-glutamine, and 10% FCS. HEK 293T, Ben-Men-1, IOMM-Lee, and Platinum-E cells were maintained at 37°C and DF-1 cells at 39°C in humidified air with 5% CO_2_.

#### Animals

Mice were bred and housed under specific-pathogen-free conditions in the animal facility of Laboratory Animal Services Center at the University of Zurich. NOD.CB17-Prkdc^scid^ (NOD-SCID) mice of 6 to 8 weeks of age were purchased from Janvier Labs. *Nestin/tv-a; Cdkn2a^-/-^; Pten^fl/fl^; Luc^LSL/LSL^* (*N/tv-a*) mice were a gift from E. Holland (Seattle, USA) and bred in-house. All animal experiments were done following the guidelines of the Swiss federal law on animal protection and were approved by the cantonal veterinary office (license no. ZH079/2021).

#### cBioPortal data collection and analysis

The transcriptional profile and the corresponding clinical information data of meningioma patients were obtained from the cBioPortal (https://www.cbioportal.org/) and the analysis is described in the supplementary methods.

#### Tissue microarray (TMA)

Tissue microarrays were stained and analyzed as described before.^32^ Additional information can be found in the supplementary methods.

#### SSTR2 overexpression in human cell lines

The SSTR2 sequence was synthesized by GenScript and cloned into pLenti-puro, including a membrane localization sequence to prevent ligand-induced receptor internalization.^33^^,^^34^ pCAG-VSVG, psPAX2 and pLenti-SSTR2-puro were used to transfect HEK 293T cells at a 1:1.75:2.25 ratio in full DMEM medium without antibiotics. Ben-Men-1, and IOMM-Lee cells were lentivirally transduced by spinoculation at 1000 g for 90 min at 32°C with a final concentration of 8 μg/ml polybrene. Twenty-four hours later, the medium was exchanged and 48 hours later, positively transduced cells were selected with 3-10 μg/ml puromycin for 3-10 days.

#### Quantitative reverse transcription polymerase chain reaction (RT-qPCR)

According to the manufacturer’s protocols, NucleoSpin RNA kit was used to extract total RNA, High-Capacity cDNA Reverse Transcription Kit was used to construct cDNA, and PowerUp™ SYBR™ Green Master Mix kit was used to amplify the cDNA template. ADP-ribosylation factor 1 was used as a housekeeping gene for mRNA level normalization. Primer sequences used for RT-qPCR were listed in the supplementary methods.

#### Octofluo labeling study

For *in vitro* labeling of tumor cells with octofluo, Ben-Men-1, and IOMM-Lee WT and SSTR2+ cells, pre-seeded at a cell density of 1 × 10^5^ cell per well in µ-Slide 8 Well chamber 12 h before the incubation, were washed with PBS twice for 5 min each and incubated with 10 nMol octofluo in PBS for 1 h at 37°C. Hoechst 33342 was added 5 min before imaging at a final dilution of 1:2000. Images were acquired using a Leica Stellaris 5 inverse confocal microscope.

#### CAR design and virus generation

The publicly available sequences of 4M5.3^35^ and E2^36^ single-chain variable fragment (scFv) as well as T2A and P2A sequences with RQR8^37^ were synthesized and cloned by GenScript. Additional expression of RQR8 was used to determine the transduction efficiency and potential purification of positively transduced T cells using anti-CD34 antibodies.^37^

For human AdFITC(E2) CAR-T cell generation, the E2 scFv was cloned into a second-generation CAR backbone vector containing CD8α hinge domain, 4-1BB and CD3ζ endodomains.^38^ The human CAR was co-expressed with the marker gene RQR8 and separated by a self-cleaving P2A peptide. The lentiviral envelope-expressing plasmid pCAG-VSVG and the packaging plasmid psPAX2 were purchased from Addgene. pCAG-VSVG, psPAX2 and CAR plasmid were used to transfect HEK 293T cells at a 1:1.75:2.25 ratio in full DMEM without antibiotics. The medium was exchanged after 24 hours. The supernatant was collected, and the lentiviruses were concentrated using PEG-it virus precipitation solution 2 days later.

The second-generation CAR construct was kindly provided by Dr. N. Okada (Osaka University, Japan) in a pMXs-Puro_CAR plasmid, encoding for a murine Igκ leader sequence followed by a scFv with a CD28-derived hinge, transmembrane, and co-stimulatory domain, including a CD3ζ domain.^39^ The scFv of the provided construct was exchanged by the scFv E2 or 4M5.3. Both murine CAR sequences (herein, AdFITC(E2) CAR and AdFITC(4M5.3) CAR) were cloned into the retroviral backbone pMSGV, which was a gift from James Kochenderfer and Steven Rosenberg,^40^ and were co-expressed with the marker gene RQR8 and separated by a self-cleaving T2A peptide. Retrovirus was produced by transfection of 10^7^ Platinum-E cells with the according plasmid and Fugene 6 Transfection Reagent at a 1:3 ratio for 24 hours. Supernatants were harvested at 48 and 72 hours after transfection and concentrated with Retro-Concentin according to manufacturer instructions.

Detailed information on human and murine CAR-T cell generation are provided in Supplementary methods.

#### Time-lapse imaging

On day 0, human IOMM-Lee cells were pre-stained using CellTrace™ CFSE Cell Proliferation Kit according to the manufacturer’s protocol and seeded at a density of 5 × 10^3^ cells per well in 100 l of complete DMEM in µ-Slide 8 Well Glass Bottom. The next day, human AdFITC(E2) CAR T cells were resuspended at a density of 2 × 10^4^ cells in 100 μl Advanced RPMI containing 10 nMol octofluo with a final concentration of 0.5 mg/ml propidium iodine and added on the human tumor cells. Several fields of interest were selected, and the imaging was performed on a Nikon MMI microdissection confocal microscope (time interval: 3 min, duration: 20 h).

#### In vitro *killing assay*

Detailed information on experimental setup are described in the supplementary methods.

#### Generation of Luciferase-expressing IOMM-Lee cells

IOMM-Lee wildtype (WT) and SSTR2 positive (SSTR2+) cells were transduced with mCherry-Luciferase virus (CMV-GFP-T2A-Luciferase; System Biosciences) as described in the supplementary methods.

#### RCAS vector construction

Plasmids (pEGH1U6, pENTR-mRFP-H1, pENTR-NLS-5SA-YAP1, RCAS-DV, and RCAS-shTp53) were kindly provided by E. Holland (Seattle, USA). Further modifications and detailed information on RCAS virus production are provided in the supplementary methods.

#### Generation and transduction of murine arachnoid cells

Mouse arachnoid tissue was gently dissected from the ventral brainstem of *N/tv-a* mice as described in the supplementary methods.

#### Induction of meningioma formation

The RCAS/tv-a somatic gene transfer system was used for meningioma induction. RCAS-producing DF-1 cells were implanted stereo-tactically into 6 to 10 weeks of age *N/tv-a* mice on day 0. For meningioma formation, on day 0, IOMM-Lee (2 × 10^5^ cells) with luciferase expression or DF-1 (4 × 10^5^ cells) were resuspended in 2 μl PBS and implanted stereo-tactically into the frontal subdural region (2.5 mm right lateral from bregma and 1.0 mm deep from the skull) of NOD-SCD mice or *N/tv-a* mice, respectively. Further information on surgery and treatment procedures are described in supplementary methods.

#### *Octofluo biodistribution* in vivo

Tumor-bearing mice were treated with 1 nMol octofluo intravenously and sacrificed at different time-points within the first 24 hours. Tumor-bearing brains and peripheral organs (spleen and liver) were cryopreserved for the following Hematoxylin and eosin (H&E) and immunofluorescence (IF) staining.

#### Bioluminescence

Bioluminescence imaging was obtained on the IVIS Spectrum *In Vivo* Imaging System. Mice with tumors expressing luciferase were anesthetized with isoflurane before peritoneal injection with 75 mg/kg body weight D-luciferin. After injection, repeat images were acquired for 5 minutes to ensure that the plateau phase of photon emission was reached. Total flux (photon·seconds^−1^) was obtained from each mouse.

#### Immunohistochemistry and IF

Detailed information and a list of antibodies used for immunohistochemistry (IHC) and IF can be found in supplementary methods.

#### Immune landscape analysis

All meningioma-bearing *N/tv-a* mice included were confirmed by IVIS and divided into treatment and control groups. The treatment group was treated with 2 × 10^6^ murine AdFITC(4M5.3) CAR T cells intratumorally and 1 nMol octofluo intravenously every 2 days. In comparison, the control group was treated with only 2 × 10^6^ murine AdFITC(4M5.3) CAR T cells intratumorally. The mice were sacrificed on day 7 after CAR-T injection and brains were isolated after a transcardial perfusion with 30 ml of ice-cold PBS. Detailed brain processing information are described in the supplementary methods.

#### Ex vivo human meningioma cell killing assay

All tissues were obtained from the Department of Neurosurgery, University Hospital Zurich, based on institutional review board approval. The freshly resected samples from meningioma were processed as described in the supplementary methods.

#### Statistical analysis

Statistical analysis was performed in GraphPad Prism 9 using student’s t-test, one-way or 2-way analysis of variance (ANOVA) as indicated and correction for multiple comparisons using the Tukey method. For survival analysis, Kaplan-Meier curves were compared, and *P* values were calculated using the log-rank test (Supplementary methods).

## Results

### High SSTR2 Expression in Meningioma is Associated with Unfavorable Outcome

**Figure 1.**
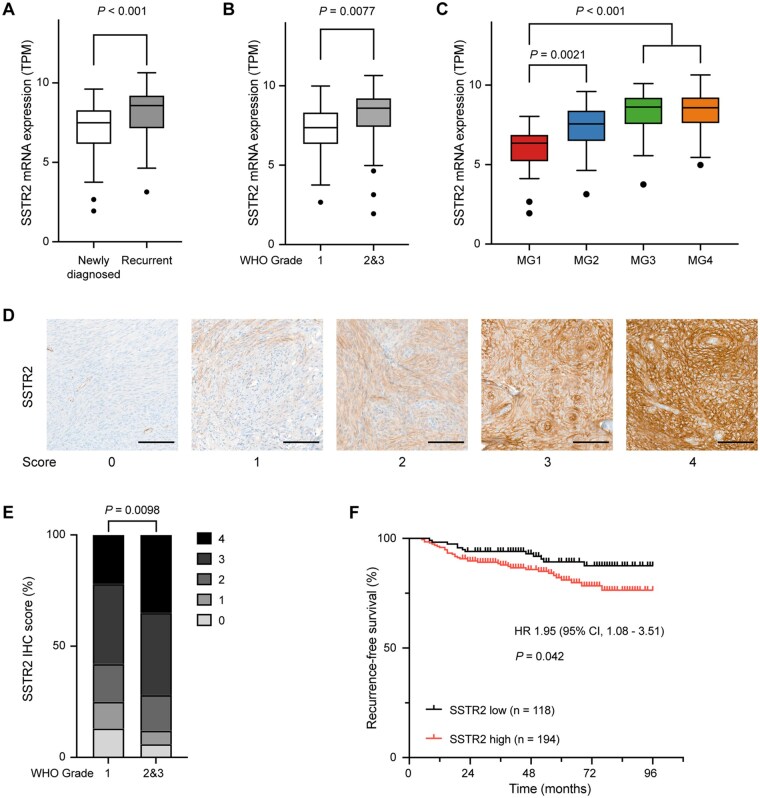
Clinical associations of SSTR2 expression in meningiomas. (A-C) SSTR2 mRNA expression levels in meningiomas (*N* = 121, *t* test).^22^ TPM, transcripts per million; MG, proposed molecular group; WHO, World Health Organization. (D) Representative images of SSTR2 immunohistochemistry (IHC) staining scores on a meningioma tissue microarray (*N* = 312). Scale bar, 100 μm. (E) SSTR2 IHC score by WHO grade (one-way ANOVA). (F) Recurrence-free survival of meningioma patients by SSTR2 expression (log-rank test). HR, hazard ratio; SSTR2 low/high, IHC score 0-2/3-4. See also Supplementary Table S1, S2, S3 and Supplementary  Figure S1.

First, we sought to determine SSTR2 abundance in clinically annotated meningioma patient samples. In a published cohort of 121 meningiomas (Supplementary Table S1),^22^ mean expression of *SSTR2* mRNA was higher in recurrent compared to newly diagnosed tumors (*t* test *P *< .001, Figure 1A). Along the same lines, *SSTR2* mRNA expression in this cohort was lower in Central Nervous System World Health Organization (CNS WHO) grade 1 compared to the clinically more aggressive CNS WHO grade 2/3 meningiomas (*t* test *P *= .0077, Figure 1B), and lower in a proposed favorable outcome meningioma molecular group (MG)1 compared to the less favorable MG2 (*t* test *P *= .0021) and MG3/4 (*t* test *P *< .001, Figure 1C). We performed a multivariable linear regression analysis using SSTR2 mRNA expression as the outcome to account for relation between recurrence status, WHO grade, and MG. MG was the only of these variables that was independently associated with SSTR2 expression (MG3/4: *P *< .001; reference: MG1), whereas WHO grade (*P *= .96) and recurrence status (*P *= .078) did not reach independent significance (R^2^ = 0.27, Supplementary Table S2).

Next, we classified SSTR2 protein abundance in 312 meningiomas for subsequent clinical association studies by employing a semi-quantitative IHC score (Figure 1D). The clinical characteristics of this cohort are summarized in Supplementary Table S3. High SSTR2 levels (IHC score 3 or 4) were observed in 59 of 77 CNS WHO grade 2/3 meningiomas (77%), but only 139 of 235 CNS WHO grade 1 meningiomas (59%, Figure 1E, one-way ANOVA *P *= .0098). These observations suggest that SSTR2 may be a suitable drug delivery target in higher grade meningiomas, for which there is an unmet clinical need. Of note, SSTR2 expression was not observed in dura or leptomeninges of autopsy patients without meningioma or other meningeal disease (Supplementary Figure S1A, B). Recurrence-free survival of patients with meningiomas classified as SSTR2 high (IHC score 3 or 4) was less favorable compared to patients with SSTR2 low (IHC score 0-2) meningiomas (Hazard ratio [HR] = 1.95, 95% confidence interval [CI] 1.08-3.51, log-rank *P *= .042, Figure 1F). Digital quantification of SSTR2 staining confirmed these observations (Supplementary Figures S1C, D).

Collectively, these data establish an association of higher SSTR2 expression in meningiomas with unfavorable outcome.

### Octofluo Binds SSTR2 on Meningioma Cell Membranes with High Affinity

Next, we set out to design a bispecific SSTR2-targeted, FITC-conjugated adapter peptide (octofluo) to direct FITC-targeted CAR-T cells against SSTR2-positive tumor cells.^41^ We refrained from using well-established SSTR2 agonists used in positron emission tomography diagnostics, because these exhibit low-affinity target interactions (dissociation constants *K_D_ ∼* 30 nM) and lead to rapid receptor internalization upon SSTR2 binding.^33^^,^^34^ By contrast, receptor *ant*agonists can be designed to achieve improved *in vivo* targeting and circumvent target internalization.^42^

**Figure 2.**
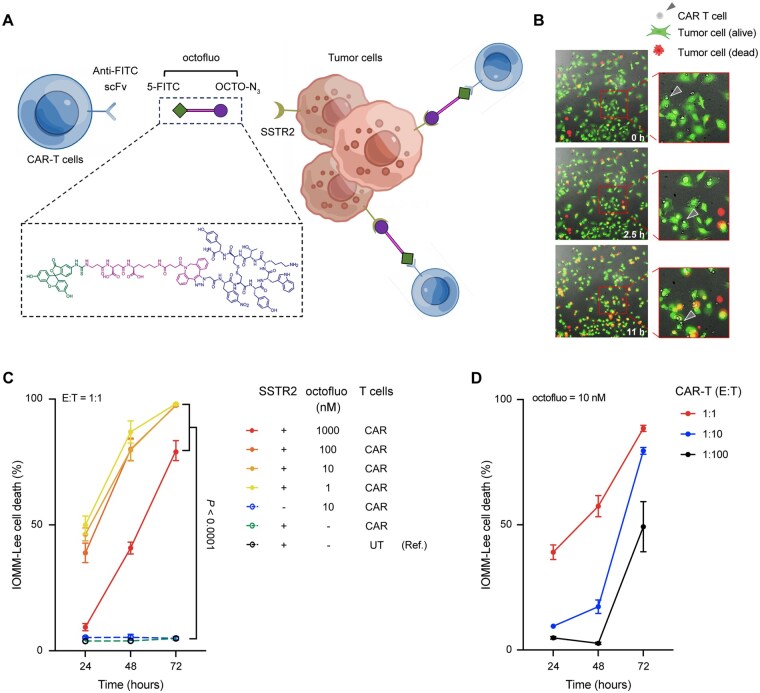
Higher grade meningioma cell lysis by AdFITC CAR-T cells plus octofluo. (A) Schematic of the AdFITC CAR-T cell plus octofluo therapeutic system. (B) Representative images of SSTR2+ IOMM-Lee cell killing by AdFITC CAR-T cells plus octofluo. Arrows, CAR-T cells (examples); live tumor cells were stained with carboxyfluorescein succinimidyl ester; dead tumor cells were stained with propidium-iodide. (C, D) IOMM-Lee meningioma cell killing assays. Reference (C): SSTR2+, no octofluo, untransduced T cells (2-way ANOVA, ****P < .0001). E:T, effector-target cell ratio. See also Supplementary Video S1 and Supplementary  Figures S2-S3.

Therefore, we composed octofluo of an octameric SSTR2 *ant*agonist sequence^42^ and further optimized tissue penetration by synthesizing a lipophilic linker to FITC (Figure 2A). To validate binding of octofluo to SSTR2 on tumor cells, we generated SSTR2+ IOMM-Lee and Ben-Men-1 human meningioma cell lines by lentiviral transduction with a modified SSTR2 cassette that yields retention of SSTR2 at the cell surface (Supplementary Figure S2A, B). This modification was required, because for unknown reasons, SSTR2 occurs *in vitro* only internalized in the cytoplasm. Immunocytology revealed membrane staining in SSTR2+ IOMM-Lee and Ben-Men-1 cells following incubation with 10 nM octofluo, whereas octofluo did not stain untransduced (WT) meningioma cells or the soma of SSTR2+ cells (Supplementary Figure S2C). These observations indicate specific, high affinity SSTR2 binding in the nanomolar range without relevant SSTR2 internalization. We concluded that octofluo fulfilled the pharmacodynamic requirements for redirecting AdFITC CAR-T cells against SSTR2+ tumor cells.

### AdFITC CAR-T Cells Lyse SSTR2+ Meningioma Cells in the Presence of Octofluo

We then sought to determine the capacity of octofluo to bridge FITC-specific CAR-T cells and SSTR2+ tumor cells. For this purpose, we generated human AdFITC CAR-T cells by lentiviral transduction using T cells from 2 healthy donors (HD27, HD28, Supplementary Figure S2D-G). Time-lapse imaging of AdFITC CAR-T cells co-incubated with SSTR2+ IOMM-Lee cells at an effector-target cell (E:T) ratio of 1:1 in the presence of 10 nM octofluo confirmed SSTR2+ tumor cell killing (Figure 2B, Supplementary Video S1). This experimental setup was then employed to determine the therapeutic range of octofluo in flow cytometry-based tumor cell killing assays of SSTR2+ IOMM-Lee and Ben-Men-1 cells (Figure 2C, Supplementary Figure S3A). At an E:T ratio of 1:1, octofluo concentrations in the range of 1-100 nM yielded similar killing efficacies in both cell lines, in the range of ∼40%-50%, ∼80%-90%, and ∼100% after 24 h, 48 h and 72 h, respectively (Supplementary Table S4). By contrast, increasing the octofluo concentration to 1000 nM reduced the killing efficacy of CAR-T cells by approximately 50% at 48 h, likely reflecting octofluo saturation of both, CAR-T cells and tumor cells. The negative control groups, employing (i) SSTR2-negative meningioma cells, (ii) absence of octofluo, or (iii) untransduced T cells, all yielded less than 5% tumor cell death. Next, we used the same experimental setup to titrate E:T ratios in the presence of 10 nM octofluo and observed killing of ∼50% of SSTR2+ IOMM-Lee or Ben-Men-1 cells at an E:T ratio of 1:100 after 72 h (Figure 2D, Supplementary Figure S3B, Supplementary   Table S5). To account for potentially unrepresentatively high SSTR2 expression due to overexpression in SSTR2+ IOMM-Lee and Ben-Men-1 meningioma cells, we performed SSTR2 siRNA transfection and repeated tumor cell killing assays (Supplementary Figure S3C). Despite reduced SSTR2 levels, we observed antigen-dependent but consistently high lysis efficiency, which was corroborated in the SSTR2-low H69 tumor cell line (Supplementary Figure S3D-G).

Collectively, these data indicate specific and efficient tumor cell killing by the octofluo-based AdFITC CAR-T cell system *in vitro*.

### Limited Efficacy of AdFITC CAR-T Cells Plus Octofluo in an Orthotopic Xenograft

In solid tumors, intratumoral (i.t.) CAR-T cell injections are deemed more efficacious and safer than systemic CAR-T cell applications.^43-46^ However, repeated applications of adapter molecules are required to redirect and activate CAR-T cells against tumor cells in a tightly regulated manner. In brain tumors, repeated i.t. injections are clinically challenging and associated with significant risk, thus requiring tumor penetration of the CAR adapter molecule following i.v. injections. To test whether octofluo fulfilled this pharmacokinetic prerequisite, we employed human SSTR2+ IOMM-Lee higher-grade meningioma cells in an orthotopic xenograft model. Indeed, a transient intratumor FITC signal was apparent at 3 to 6 hours after i.v. injection of 1 nMol octofluo and this signal was dim at 12 hours and no longer detected at 24 hours (Supplementary Figure S4A). At the same time, no FITC signal was observed in peripheral organs (Supplementary Figure S4B).

**Figure 3.**
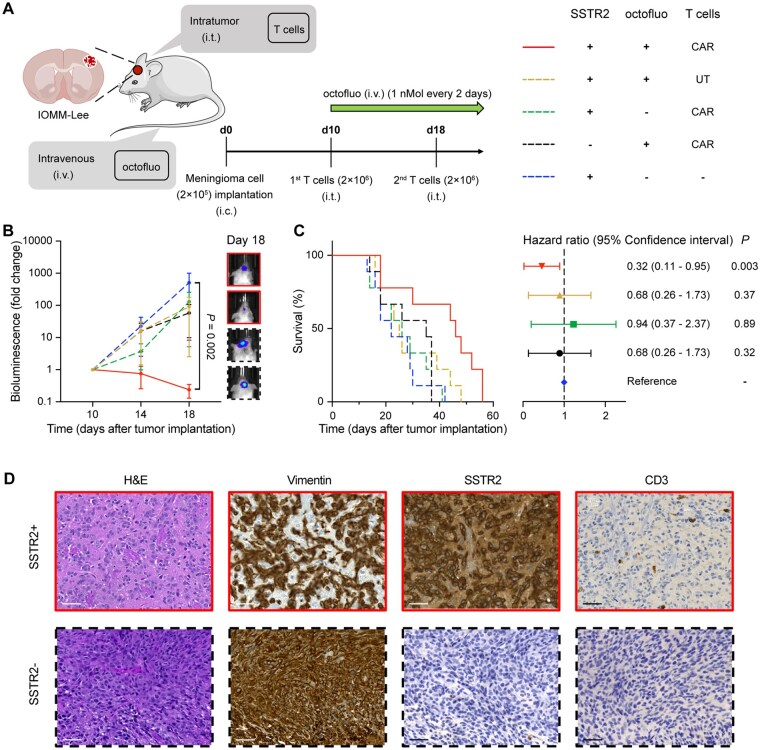
Preclinical trial of AdFITC CAR-T cells plus octofluo in the IOMM-Lee higher grade meningioma xenograft. (A) Experimental setup. SSTR2+ or SSTR2-IOMM-Lee meningioma cells were implanted orthotopically in the subdural space of *NOD-SCID* mice. (B) Growth of orthotopically implanted IOMM-Lee meningiomas assessed *in vivo* by bioluminescence imaging (*N* = 5/group). Representative images of SSTR2+ and SSTR2- tumors, as iindicated. Experimental group annotations are indicated in panel (C), Reference: SSTR2+, PBS i.v./i.t. (2-way ANOVA). (C) Survival of mice harboring SSTR2+ or SSTR2- IOMM-Lee orthotopic meningioma xenografts, treated as indicated (*N*=9/group). Reference: SSTR2+, PBS i.v./i.t. (log-rank). E:T, effector to target cell ratio; CAR, FITC-specific chimeric antigen receptor T cells; UT, untransduced T cells. (D) Immunohistochemistry of SSTR2+ and SSTR2- IOMM-Lee xenografts treated with AdFITC CAR-T cells plus octofluo. Tumors were isolated one week after treatment ­initiation. Scale bars, 100 μm. See also Supplementary Figures S4-S6.

Next, we employed the IOMM-Lee orthotopic meningioma xenograft model to also test whether a clinically feasible treatment schedule combining 2 i.t. AdFITC CAR-T cell injections and every 2 days intravenous (i.v.) octofluo applications would exert relevant anti-tumor activity (Figure 3A). Compared to i.t. and i.v. injections of PBS, this treatment schedule led to tumor regression on serial *in vivo* bioluminescence imaging by day 7 after treatment initiation (2-way ANOVA *P *= .002), whereas no growth reduction was observed in the 3 control groups, including (i) AdFITC CAR-T cells i.t. with octofluo i.v. in SSTR2- tumors (2-way ANOVA *P = *.19), (ii) AdFITC CAR-T cells i.t. without octofluo in SSTR2+ tumors (2-way ANOVA *P = *.54), or (iii) untransduced T cells i.t. with octofluo i.v. in SSTR2+ tumors (2-way ANOVA *P = *.54, Figure 3B, Supplementary Figure S5). Along the same lines, 2 i.t. AdFITC CAR-T cell injections combined with intravenous (i.v.) octofluo every 2 days also prolonged survival of SSTR2+ IOMM-Lee meningioma-bearing mice compared to treatment with i.t. and i.v. PBS (HR = 0.15, CI 0.11-0.95, log-rank *P = *.003), although we did not observe long-term survival or cures of mice. As expected, no survival benefit was observed in the control groups upon treatment with (i) AdFITC CAR-T cells i.t. with octofluo i.v. in SSTR2- tumors (log-rank *P = *.32), (ii) AdFITC CAR-T cells i.t. without octofluo in SSTR2+ tumors (log-rank *P = *.89), or (iii) untransduced T cells i.t. with octofluo i.v. in SSTR2+ tumors (log-rank *P = *.37, Figure 3C).

Hematoxylin and eosin staining as well as IHC on day 7 after the initiation of treatment with AdFITC CAR-T cells i.t. and octofluo i.v. revealed reduced tumor cell density and corresponding maintenance of human CAR-T cells in SSTR2+, but not in SSTR2- IOMM-Lee meningioma xenografts (Figure 3D). Reduced tumor cell density or persistence of CAR-T cells were neither observed in control groups, including SSTR2+ meningiomas treated with (i) PBS i.t. and i.v., (ii) untransduced T cells i.t. with octofluo i.v., or (iii) AdFITC CAR-T cells i.t. without octofluo (Supplementary Figure S6).

These data indicate that orthotopic targeting of SSTR2+ meningioma cells by combined AdFITC CAR-T cells i.t. and octofluo i.v. is feasible and specific. However, the observed anti-tumor efficacy in the immunocompromised IOMM-Lee orthotopic xenograft model was not durable.

### In Situ *Induction of Higher Grade Meningiomas by RCAS/tv-a Somatic Gene Transfer*

The initiation of host anti-tumor immunity can contribute to the efficacy of CAR-T cell therapies.^47^ Therefore, we reasoned that the limited efficacy of AdFITC CAR-T cells i.t. with octofluo i.v. in the IOMM-Lee xenograft model may have resulted from a lack of adaptive host T cell immunity. To test this hypothesis, we sought to also explore the efficacy of AdFITC CAR-T cells with octofluo in the context of an intact immune system. For this purpose, we employed the RCAS/tv-a somatic gene transfer system to genetically engineer a murine model of *Nf2-*deficient higher-grade meningioma.

**Figure 4.**
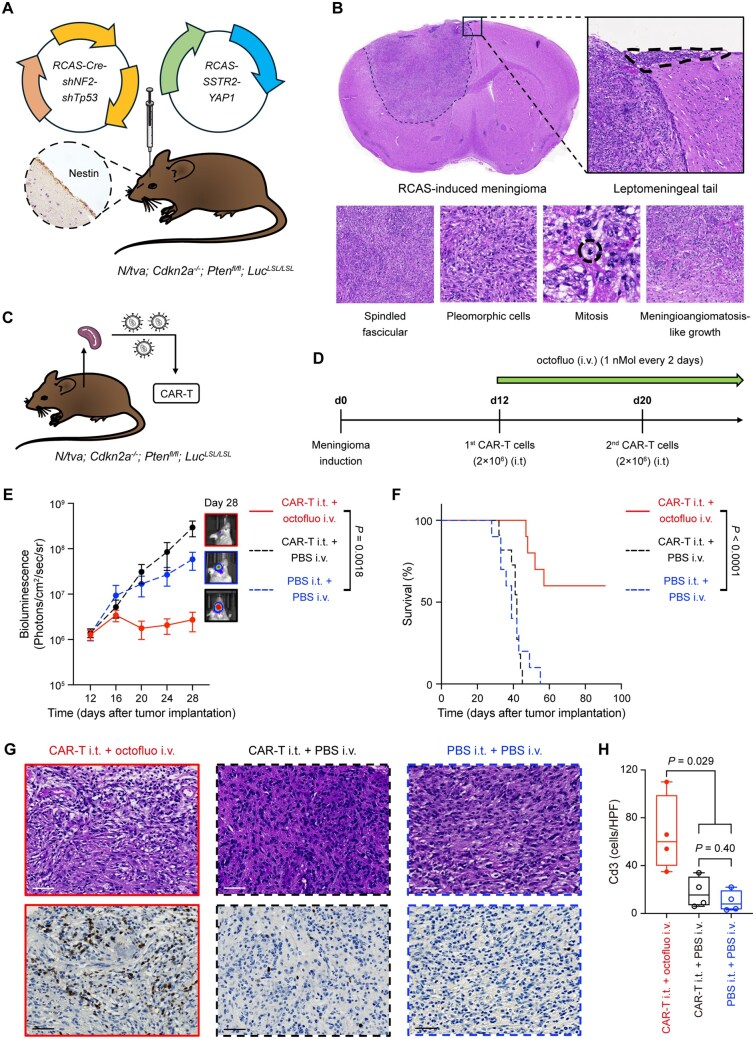
Preclinical trial of AdFITC CAR-T cells plus octofluo in immunocompetent, genetically engineered murine meningiomas. (A) Schematic of genetic engineering of murine meningiomas utilizing the RCAS/tv-a somatic gene transfer system. Indicated transgenic mice express the RCAS entry receptor tv-a under control of the Nestin gene promoter. Nestin expression in the murine arachnoidea was confirmed by immunohistochemistry (inlay). CAR, FITC-specific chimeric antigen receptor T cells; UT, untransduced T cells. (B) Representative H&E staining of genetically engineered murine meningiomas. (C) Schematic of AdFITC CAR-T cell production by lentiviral transduction. (D) Timeline of AdFITC CAR-T cell plus octofluo therapy as conducted in (E) and (F). (E) Growth of murine meningiomas assessed *in vivo* by bioluminescence imaging (*N* = 5/group) and representative images (inlays). Reference: PBS i.v./i.t. (2-way ANOVA). (F) Survival of mice harboring genetically engineered murine meningiomas and treated as indicated (*N* = 10/group). Reference: PBS i.v./i.t. (log-rank). (G) H&E and anti-Cd3 immunohistochemistry of murine meningiomas treated as indicated. Scale bars, 100 μm. (H) Quantification of Cd3+ cells one week after i.t. injection of CAR-T cells or PBS (*N*=4/group, *t* test, experimental groups are indicated by color code). See also Supplementary Figures S7-S9.

We utilized *N/tv-a; Cdkn2a^-/-^; Pten^fl/fl^; Luc^LSL/LSL^* mice, which express the RCAS entry receptor tv-a under control of the Nestin gene promotor,^48^ thus enabling the specific transduction of Nestin-expressing cells. Nestin expression by arachnoidal cells was reported previously^49^ and was confirmed by IHC (Figure 4A). Two RCAS vectors were then cloned for *in situ* transformation of arachnoidal cells to model genetic features of higher grade meningiomas^22^^,^^50^^,^^51^: Expression of a constitutively active mutant of the yes-associated protein 1 gene *(5SA-Yap1)* was forced to model down-stream effects of *Nf2* loss-of-function^49^ and was linked to SSTR2 by a P2A self-cleaving peptide; expression of Cre recombinase by the second vector induced *Pten* knockout and activation of a Luciferase gene cassette and was combined with *shNf2* and *shTp53* gene silencing cassettes (Figure 4A, Supplementary Figure S8A-C). Injection of both vectors into the subdural space of adult *N/tv-a; Cdkn2a^-/-^; Pten^fl/fl^; Luc^LSL/LSL^* mice induced tumors that exhibited a pathognomonic leptomeningeal tail sign as well as histopathologic features of anaplastic meningioma, including the presence of a variable biphasic spindle cell appearance with occasional nuclear pleomorphism and enlarged nucleoli, areas of pattern-less growth, macronucleoli, pleomorphic nuclei, and meningioangiomatosis-like growth and invasion (Figure 4B). In addition to this frank anaplasia, each of these meningiomas would be graded as CNS WHO grade 3 due to the presence of homozygous deletion of *Cdkn2a.*^18^ This molecular alteration is grade defining within meningiomas. Finally, we have quantified Ki67 staining of the whole tumor area of RCAS-induced murine meningiomas, reflecting the percentage of cells in the mitotic cycle in the range of 14.85-42.63/mm^2^, thus fulfilling a third independent criterion for CNS WHO grade 3, ie a mitotic index of >12.5/mm2 (Supplementary Figure S7).^18^

The main tumor mass is continuous with the leptomeninges, often forming a leptomeningeal tail (Figure 4B). The continuity of the tail with the main tumor mass is characterized by histologically similar cells that are syncytial and blend smoothly into the adjacent leptomeninges. This is akin to carcinoma arising from an adjacent intraepithelial (*in situ*) neoplasm in lower organs. Overall, the histologic features of these tumors are consistent with a leptomeningeal origin.

In summary, this adult genetically engineered murine model of higher-grade meningioma developed from the own arachnoidal cells of *N/tv-a; Cdkn2a^-/-^; Pten^fl/fl^; Luc^LSL/LSL^* mice in the context of a fully intact immune system and exhibited high similarity to its human counterpart.

### AdFITC CAR-T Cells plus Octofluo Cure Murine Higher Grade Meningiomas

As a next step to exploring the efficacy of octofluo-targeted CAR-T cell therapy in the context of an intact host immune system, we generated murine CAR-T cells through retroviral transduction of T cells isolated from the spleens of *N/tv-a; Cdkn2a^-/-^; Pten^fl/fl^; Luc^LSL/LSL^* mice (Figure 4C, Supplementary Figure S8D, E) and conducted efficacy testing by *ex vivo* killing assays with SSTR2+ murine transformed arachnoidal cells (Supplementary Figure S8F, G).

We then conducted a preclinical trial in genetically engineered meningioma-bearing mice to compare the efficacy of AdFITC CAR-T cells i.t. with or without octofluo i.v. every other day to meningioma-bearing mice treated with i.t. and i.v. PBS (Figure 4D). Tumor growth was confirmed by *in vivo* bioluminescence imaging prior to treatment initiation and was longitudinally assessed (Supplementary Figure S9). Compared to PBS-treated controls, AdFITC CAR-T cells i.t. inhibited tumor growth in combination with octofluo i.v. (2-way ANOVA *P = *.0018), but not without octofluo (2-way ANOVA *P = *.064, Figure 4E, Supplementary Figure S9). Along the same lines, survival of meningioma-bearing mice was prolonged only in the combined AdFITC CAR-T cell i.t. plus octofluo i.v. treatment arm (HR = 0.08, 95% CI 0.02-0.29, log-rank *P *< .0001), including 6 of 10 treated mice exhibiting long-term survival (Figure 4F).

Histopathologic workup on day 7 after treatment initiation revealed an increase in CD3+ T cells on H&E stainings in meningiomas from 10 cells/high power field (HPF) in the PBS group to 66 cells/HPF in the co-treated with AdFITC CAR-T cells i.t. with octofluo i.v. group (*t* test *P = *.029), whereas no increase in T cell numbers occurred in tumors treated with CAR-T cells alone (13 cells/HPF, *t* test *P = *.40, Figure 4G, H).

Collectively, these data suggest potent and specific efficacy of our AdFITC CAR-T cell system in the context of an intact immune system.

### AdFITC CAR-T Cells plus Octofluo Induce an Endogenous Immune Response in Murine Higher Grade Meningiomas

**Figure 5.**
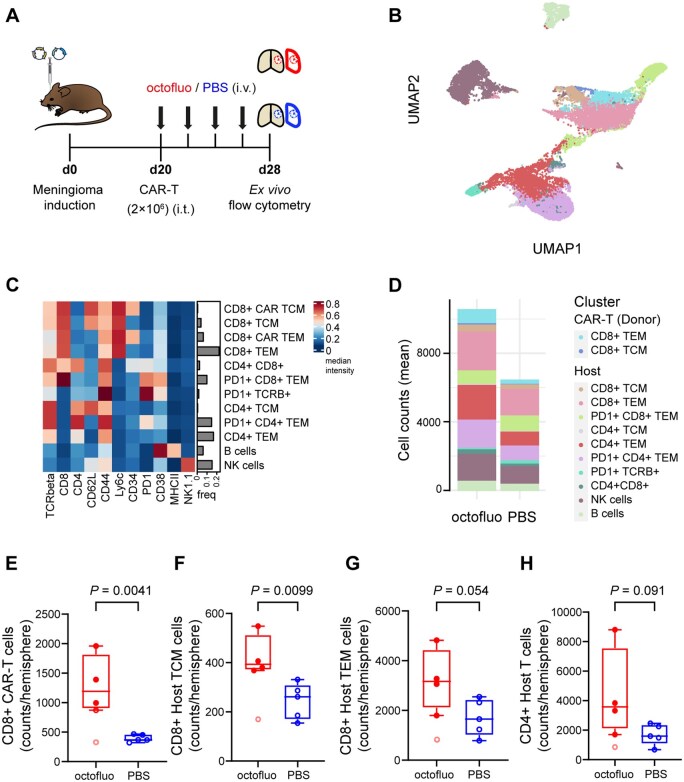
Tumor microenvironment multi-dimensional flow cytometry of genetically engineered murine meningiomas treated with AdFITC CAR-T cells. (A) Experimental setup. Murine meningiomas were treated with i.t. AdFITC CAR-T cells plus i.v. octofluo or PBS (*N* = 5/group). (B) Uniform Manifold Approximation and Projection (UMAP) visualization of immune cell populations indicated in panel (D) in meningioma bearing hemisphere (*N* = 5/group). (C) Heatmap of immune cell populations identified by indicated cell and phenotype marker expression. (D) Mean counts of cell populations indicated in panel (*n* = 5/group). (E-H) Cell counts of Cd8+ CAR-T cells (E), CD8+ central memory endogenous T cells (F), CD8+ effector memory endogenous T cells (G), and CD4+ endogenous T cells (H, *N* = 4-5/group, *t* test). One outlier in the experimental group (open circle) was not included in the statistical analysis for the lack of CAR-T cell expansion. See also Supplementary Figures S10-S12.

Next, we sought to further substantiate the effects of our AdFITC CAR-T cell system on the composition of the tumor microenvironment of murine meningiomas by high-dimensional flow-cytometry. Mice with established meningiomas were co-treated with AdFITC CAR-T cells i.t. on day 1 and with octofluo or PBS i.v. every other day for 1 week, then tumor-bearing hemispheres and meninges were harvested, and high dimensional flow cytometry was performed (Figure 5A).

High dimensional analysis of the lymphoid compartment on day 7 after treatment initiation revealed the presence of different CD4^+^ and CD8^+^ T cell clusters, including central memory T cells and effector memory T cells as well as B cells (CD38^+^MHCII^+^) and NK cells (NK1.1^+^) (Figure 5B, C). Additionally, co-expression of CD34 (RQR8) by AdFITC CAR-T cells allowed us to differentiate between donor-derived CAR-T cells and endogenous T cells (Figure 5C).^37^ Octofluo i.v. treatment induced a significant expansion of several lymphoid cell subsets in tumor-bearing hemispheres (Figure 5D). In the i.v. octofluo-treated compared to the i.v. PBS-treated group, CD8+ CAR-T cells expanded by >3-fold in 4/5 tumor bearing hemispheres (octofluo vs PBS mean counts [range] = 1305 [872-1960] vs 396 [322-468], *t* test *P *= .0041, Figure 5E), comprising mainly effector memory T cells (Fig. S10A, B). Within hemispheres from the octofluo-treated group that exhibited CAR-T cell expansion, there was also an approximately 2-fold expansion of host-derived central memory CD8+ T cells (octofluo vs PBS mean counts [range] = 426 [368-548] vs 243 [154-331], *t* test *P *= .0099, Figure 5F), but not of host-derived effector memory CD8+ T cells (*t* test *P *= .054, Figure 5G, Supplementary Figure S10C, D). We also noted an expansion of CD4+ T cells by trend (octofluo vs PBS mean counts [range] = 4417 [1701-8802] vs 1696 [683-2459], *t* test *P *= .091, Figure 5H), which was driven mainly by PD1+ effector memory subpopulations (Supplementary Figure S10E-G), indicating activation of CD4+ cells. No effect of octofluo treatment on B cells or NK cells was noted (Supplementary Figure S10H, I).

The myeloid compartment was analyzed in the same setup, i.e. treated with intratumor CAR-T cells with or without octofluo for 7 days. Microglia constituted the vast majority of myeloid cells irrespective of the treatment arm, whereas an expansion of classical dendritic cells type 2 (*t* test *P *= .028) and, by trend, of border-associated macrophages (*t* test *P *= .065), was confined to tumors of octofluo-treated mice (Supplementary Figures S11 and S12).

In summary, *ex vivo* flow cytometry demonstrated that octofluo i.v. induced an expansion of AdFITC CAR-T cells and induced an accompanying endogenous T cell and myeloid cell response in genetically induced murine higher grade meningiomas.

### Ex Vivo Lysis of Human Meningioma Cells by AdFITC CAR-T Cells plus Octofluo

**Figure 6.**
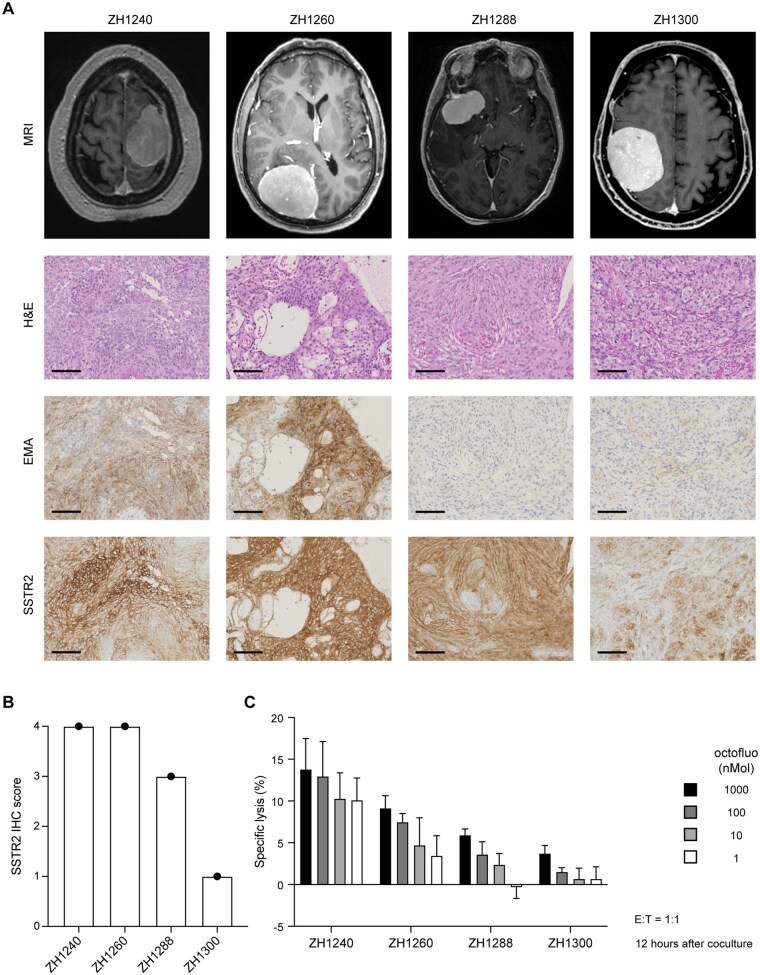
Patient meningioma cell lysis by AdFITC CAR-T cells plus octofluo *ex vivo*. (A) Representative MRI, H&E and immunohistochemistry stainings of human patient meningiomas. Scale bars, 100 μm. (B) SSTR2 IHC score of human patient meningiomas. (C) Meningioma *ex vivo* cytotoxicity assay of freshly dissociated human meningiomas incubated with AdFITC CAR-T cells and indicated concentrations of octofluo. E:T, effector-target ratio.

In patient meningiomas, SSTR2 expression is more variable and heterogeneous compared to preclinical models. Therefore, we conducted *ex vivo* killing assays in 4 freshly dissociated human meningiomas as a final step to exploring the feasibility of octofluo-directed AdFITC CAR-T cell therapy. Figure 6A summarizes presurgical magnetic resonance imaging scans and representative histopathologic workup. Semi-quantitative IHC scoring indicates SSTR2 expression (Figure 6B). Dissociated tumor cells were gated by membrane SSTR2 to limit confounding by non-neoplastic cells and receptor internalization and were co-incubated in the continuous presence of different octofluo concentrations and human AdFITC CAR-T cells at an ET of 1:1. Specific cell lysis (%) was defined as excess dead cells compared to cells co-incubated with AdFITC CAR-T cells, but without octofluo. Cell lysis was assessed at the earliest possible time-point after 12 h of co-incubation to limit bias from dissociation-induced cell death and determined up to 13% excess dead cells by flow cytometry in this proof-of-concept experiment (Figure 6C).

## Discussion

Although several CAR-T cell products have been approved for patients with hematologic malignancies,^52^^,^^53^ efficacy in solid tumors is confined to anecdotic reports.^9^^,^^43^^,^^54-56^ Reasons for this limited efficacy of CAR-T cells in solid tumors include the presence of microanatomic physical and immunosuppressive barriers that cause poor trafficking and exhaustion of CAR-T cells as well as antigen heterogeneity and antigen loss.^10^^,^^57^ On the other hand, potentially lethal toxicity from overshooting tumor cell lysis or off-tumor activity can challenge the development of CAR-T cell therapies, but efficacy appears to be the more prominent challenge in the development of CAR-T cell therapies for solid tumors.^58^^,^^59^

This report demonstrates the feasibility of close regulation and effective meningioma cell killing utilizing an adapter CAR-T cell system that was optimized for the treatment of solid tumors. Our system employs the tissue penetrating bispecific small molecule adapter peptide octofluo as an on-switch to bridge FITC-targeting CAR-T cells and the tumor cell-associated receptor SSTR2. Titration of adapter molecules can limit toxicity from overshooting CAR-T cell activity, whereas our study supports that metronomic adapter application schedules may on the other hand enhance CAR-T cell effectivity by counteracting exhaustion.^60^

Although our observation of a short tumor persistence time of octofluo suggests favorable pharmacokinetic properties for tight AdFITC CAR-T cell regulation in patients, this might as well challenge the maintenance of AdFITC CAR-T cell activity due to insufficient activation. We have addressed this potential caveat by limiting octofluo administrations *in vivo* to every other day and yet observed relevant anti-tumor activity of AdFITC CAR-T cells. This is in line with reports that metronomic CAR-T cell activation can circumvent CAR-T cell exhaustion and induce T cell memory formation.^8^^,^^61^ Identifying optimum clinical dosing schedules that account for the balance between CAR-T activity and exhaustion require further investigation and may require portable, programmable, continuous intravenous infusion system. Alternative strategies, eg subcutaneous delivery or half-life extension approaches, would compromise the key safety advantage of Octofluo that is conveyed by its short half-life and tumor persistence time, ie regulation of CAR-T activity per i.v. administration.

Our study also addressed accumulating evidence pointing toward a critical role of endogenous lymphocyte activation for the effectiveness of CAR-T cell therapies in solid tumors.^58^^,^^59^ We employed the RCAS/tv-a somatic gene transfer system to expand on a Yap-driven, genetically engineered murine meningioma model^49^ by introducing additional vector cassettes to mimic the molecular phenotype of higher grade meningioma.^22^^,^^23^ The resulting tumors were histopathologically indistinguishable from their human counterparts and exhibited a similar myeloid cell-dominant, lymphocyte-depleted immune phenotype. Our AdFITC CAR-T cell system cured a substantial proportion of these meningioma-bearing mice and multidimensional flow cytometry revealed CAR-T cell expansion as well as endogenous T cell infiltration and activation. By contrast, the same treatment schedule led to only a minor prolongation of survival in a similarly aggressive orthotopic xenograft model of higher-grade meningioma in NOD SCID mice that lack lymphocytes. This observation underscores that host immunity likely contributes to the efficacy of CAR-T cell therapies.

In solid tumors, the success of CAR T cell therapy is probably dependent on its ability to induce antigen spreading to expand the immune response toward secondary epitopes and prime endogenous T cells toward tumor antigens.^62^ Similar to our observations in the immune competent meningioma model, preclinical and clinical studies of CAR T cell therapy against CNS tumors demonstrated an increase in absolute host T cell numbers within the tumor microenvironment and an activated phenotype of host CD4+ and CD8+ T cells, translating into a higher tumor cell killing capacity of both, host and CAR-T cells.^63^

Adapter CAR-T cell systems employ “universal” CAR-T cells that can be activated simultaneously by various adapter molecules that differ in their target specificity but share an identical CAR recognition site. Therefore, octofluo could be used in combination with other FITC-conjugated adapter molecules to induce activity simultaneously through several antigens to enhance effectiveness and to evade antigen loss, a key mechanism of resistance to CAR therapies.^14^^,^^64^^,^^65^ Of note, adapter CAR-T cell concepts that have been developed for this purpose to treat hematologic malignancies often employ conjugates based on antibodies^65^ or single chain variable fragments.^11^^,^^12^ Such systems are however not suited for the treatment of solid tumors, because limited tissue penetration of antibodies is not ideal and their long half-life requires additional means beyond adapter titration to regulate CAR activity and to circumvent CAR-T cell exhaustion from continuous activation. Therefore, small molecule adapters have been proposed to overcome the pharmacokinetic limitation of antibody-based CAR adapters^14^ and to enable a more timely regulation of CAR-T cell activity.^3-6^^,^^60^^,^^66^

Similar to our experimental *in vivo* designs, the risk of on-target off-tumor activity should be addressed clinically by i.t. injection of AdFITC CAR-T cells to limit CAR-T cell circulation. Local application of CAR-T cells was also associated with less toxicity and signs of efficacy in brain tumor patients.^43^^,^^45^^,^^54^^,^^55^ The toxicity of AdFITC CAR-T cells is currently being evaluated in combination with FITC-folate in a phase I clinical trial in children with osteosarcoma (NCT05312411). Indirect evidence from diagnostic scans and radionuclide therapeutic approaches utilizing SSTR2 agonist binding moieties suggests intratumor accumulation rather than toxicity to healthy tissues,^29^^,^^30^^,^^67^^,^^68^ but even a limited anti-host immune response may have devastating consequences. Preclincal and clinical reports suggest that locoregional CAR-T cell application is a suitable strategy to mitigate this risk.^69^^,^^70^

We have focused this report on meningioma, because these tumors are paradigmatic for high SSTR2 expression levels and because there is an unmet medical need for the substantial proportion of meningioma patients in whom other local therapeutic options have been exhausted.^24^ Another recent study demonstrated effectiveness of CAR T cells in xenograft mouse meningioma models.^71^ Beyond meningiomas, our AdFITC CAR-T cell system is also applicable to other SSTR2-expressing tumor types, including the rare group of neuroendocrine tumors^41^^,^^72^ and several types of malignant neoplasms with less consistent SSTR2 expression.^73-76^ Along these lines, radioligand therapies that rely on conjugates with the highly specific SSTR2 agonist DOTATATE have been investigated clinically. However, cell lysis by AdFITC CAR-T cells suggests potentially higher effectivity compared to radioligand approaches, because ionizing irradiation induces growth arrest at the most, but rarely tumor shrinkage.^77^ Moreover, radioligands convey a risk of kidney toxicity that can be difficult to manage.^78^

The present study has limitations that require attention. Direct experimental assessment of on-target off-tumor toxicity was precluded in our preclinical models, because Octofluo does not bind murine SSTR2 due to limited sequence orthology. Future preclinical safety studies employing SSTR2+ models—eg tumor organoids or organotypic slice cultures—will be an important step toward clinical translation. Moreover, the utilized genetically engineered murine meningioma model can only be induced at a certain timepoint during the lifespan of mice and did therefore not allow for tumor rechallenging by syntransplantation to formally prove endogenous immunity. Future studies employing inbred transgenic mouse strains that permit syntransplantation of genetically engineered meningioma cells into lymphocyte-deficient hosts or utilizing selective immune cell depletion strategies that do not affect adoptively transferred CAR-T cells, will be important to dissect the relative contributions to tumor control of direct CAR-T cell cytotoxicity and secondary endogenous immune activation. Molecularly, our genetic model reflects a rare, but clinically relevant type of meningioma. There is also a possibility that the success of the AdFITC CAR-T cell system in the genetic model was in part due to the expression of xenoantigens in the mouse background. Along the same lines, there is currently no clinical evidence addressing whether octofluo alone is capable of inducing an immune response and if repeated octofluo injections can result in anti-drug immunity that may induce resistance.^79^ Methods to predict the immunogenicity of peptide-based drugs include computational tools, eg for epitope prediction of MHC-II binding, or dendritic cell loading assays. However, the correlation of immunogeneicity observed in pre-clinical models and human study participants appears to be limited.^79^ Adaption of the amino acid sequence to eliminate immunogenic epitopes, or deimmunization may be considered to mitigate the risk of anti-drug immunity. Finally, octofluo-independent, rapid SSTR2 internalization *ex vivo* due to ligand binding^33^^,^^34^ and rapid spontaneous cell death of freshly dissected meningiomas constrained the feasible timeframe of killing assays in freshly dissected meningiomas, thus limiting insights into the killing capacity of our AdFITC CAR-T cell system in the context of physiologic SSTR2 expression levels in patient tumors. Moreover, only modest tumor cell killing in *ex vivo* patient meningioma samples compared to our *in vitro* and *in vivo* models may in part reflect artificially elevated SSTR2 surface availability due to ectopic overexpression rather than physiologic receptor levels. Future research will focus on more accurately recapitulating clinically relevant receptor biology and tissue architecture, eg by employing organotypic slice cultures or organoids. Such models would improve the generalizability of our findings beyond the limited number of cell lines investigated here and allow broader validation across the spectrum of SSTR2-expressing tumor types.

Overall, this report supports the feasibility of small molecule adapters to improve the efficacy and safety of CAR-T cell therapies in solid tumors.

## Supplementary Material

vdag186_Supplementary_Data

## Data Availability

All data are available in the main text or supplementary materials. Further information and requests for resources and reagents should be directed to and will be fulfilled by the Lead Contact, Hans-Georg Wirsching (hans-georg.wirsching@usz.ch).
