# Supplementary material for: A fluorescein-conjugated somatostatin receptor antagonist adapter for chimeric antigen receptor T cell therapy of meningioma
Source: Neurooncol Adv. 2026 Jul 21;8(1):vdag186. doi: 10.1093/noajnl/vdag186 (PMC13450690; doi:10.1093/noajnl/vdag186)
Supplement: Supplementary file 1 [file vdag186_supplementary_data.zip › NOA_AdFITC_Suppl_clean.pdf]

Supplementary Information

A fluorescein-conjugated small molecule adapter peptide for chimeric antigen receptor T cell therapy of meningioma

Table S1. Patient characteristics (Nassiri et al. 2021)……………………………….2
Table S2. Multivariable linear regression analysis of SSTR2 mRNA expression) …………..…………………………………………..…..…………………………….3
Table S3. Patient characteristics (Padevit et al. 2023)……………………………4
Tables S4 and S5. Efficacy of in vitro CAR-T cell killing assays………………………5

Figure S1. SSTR2 expression in healthy tissue and clinical association in meningiomas. ………………………………………………………………………………6
Figure S2. Generation of human SSTR2+ cell lines and AdFITC CAR-T cells……..…7
Figure S3. AdFITC(E2)-CAR T-cells cytotoxicity against Ben-Men-1 and low SSTR2-expressing cell lines. ……………………………………………….………………9
Figure S4. Biodistribution of octofluo in orthotopic IOMM-Lee meningiomas and peripheral organs. ………………………………..…………………………..11
Figure S5. Bioluminescence imaging of orthotopic IOMM-Lee meningiomas……..12
Figure S6. Histopathologic workup of orthotopic IOMM-Lee meningiomas……..…15
Figure S7. Mitotic index of orthotopic RCAS-induced meningiomas……..........……16
Figure S8. Genetic engineering of meningiomas utilizing the RCAS/tv-a somatic gene transfer system and generation of murine AdFITC CAR-T cells……..........……17
Figure S9. Bioluminescence imaging of genetically engineered meningiomas……...19
Figure S10. Host lymphocyte populations in murine meningiomas treated with AdFITC CAR-T cells……………………………………………………………………..20
Figure S11. Myeloid tumor microenvironment multi-dimensional flow cytometry of genetically engineered murine meningiomas treated with AdFITC CAR-T cells……22
Figure S12. Host myeloid populations in murine meningiomas treated with AdFITC CAR-T cells……………………………………………………………………..24

Supplementary References……..…………………………………………………25

Table S1. Patient characteristics *(Nassiri et al., 2021). Related to Figure 1.*

	N = 121
Age: Median (range)	58 (24 - 86)
WHO grade: N (%)	
1	59 (49)
2	43 (36)
3	19 (15)
Molecular group (MG): N (%)	
MG1 (Immunogenic)	17 (14)
MG2 (Benign *NF2* wildtype)	32 (26)
MG3 (Hypermetabolic)	43 (36)
MG4 (Proliferative)	29 (24)
Recurrence at any time: N (%)	
Yes	64 (53)
No	57 (47)

Abbreviations: WHO, World Health Organization; MG, Molecular group.

Table S2. Multivariable linear regression analysis of SSTR2 mRNA expression. *Related to Figure 1.*

Variable	β coefficient (95% CI)	P value
Intercept [MG1, Newly diagonsed, G1]	5.739 (5.011 - 6.466)	<0.001
Molecular Group [MG2]	1.409 (0.507 - 2.312)	0.002
Molecular Group [MG3]	2.131 (1.233 - 3.028)	<0.001
Molecular Group [MG4]	2.048 (0.992 - 3.104)	<0.001
Tumor Recurrence [Recurrent]	0.554 (-0.064 - 1.172)	0.078
WHO Grade [G2&3]	0.015 (-0.629 - 0.659)	0.963

Model summary

Metric	Value
R^2	0.27
Adjusted R^2	0.236
F-statistic	8.061
P (model)	<0.001
n	115
Condition Number	8.34

Table S3. Patient characteristics *(Padevit et al., 2023). Related to Figure 1.*

		N = 312
Age (years):		
		52 (27 - 89)
Gender: N (%)		
	Male	100 (32)
	Female	212 (68)
Histology: N (%)		
	Fibrous	22 (7)
	Meningiothelial	103 (32)
	Angiomatous	3 (1)
	Transitional	82 (26)
	Psammomatous	3 (1)
	Secretory	7 (2)
	Microcystic	6 (2)
	Lymphoplasmacyte-rich	11 (4)
	Atypical	63 (20)
	Chordoid	3 (1)
	Clear cell	2 (1)
	Anaplastic	5 (2)
	Rhabdoid	2 (1)
WHO grade (%)		
	1	235 (75)
	2	70 (23)
	3	7 (2)
Recurrence (%)		
	Yes	46 (15)
	No	266 (85)
SSTR2: IHC Score (%)		
	0	34 (11)
	1	32 (10)
	2	52 (17)
	3	114 (37)
	4	80 (25)

Abbreviations: WHO, World Health Organization. IHC, immunohistochemistry.

Table S4. Efficacy of in vitro CAR-T cell killing assay. *Related to Figures 2 and S3.*

		1um	100nm	10nm	1nm	Negative Ctrl (i)	Negative Ctrl (ii)	Negative Ctrl (iii)
		Mean±SD						
IOMM-Lee	24h	9.40±0.77	38.90±2.11	46.23±1.40	49.95±1.96	5.29±0.20	3.88±0.33	3.82±0.21
	48h	40.83±1.28	80.05±2.42	79.73±2.32	86.95±2.39	5.36±0.65	3.91±0.18	3.95±0.32
	72h	79.53±2.16	97.50±0.16	97.65±0.32	98.13±0.11	4.93±0.36	4.87±0.18	4.88±0.09
Ben-Men-1	24h	6.36±0.41	31.43±1.80	37.03±2.48	40.80±1.78	4.69±0.16	3.85±0.19	3.81±0.21
	48h	27.40±1.80	66.83±5.08	77.80±3.57	79.98±4.51	3.91±0.16	4.14±0.44	3.74±0.68
	72h	74.43±2.45	95.40±0.45	95.95±0.27	97.50±0.37	4.94±0.25	4.61±0.14	4.73±0.62

Table S5. Efficacy of in vitro CAR-T cell killing assay. *Related to Figures 2 and S3.*

		E:T=1:1	E:T=1:10	E:T=1:100
		Mean±SD		
IOMM-Lee	24h	39.05±1.59	9.49±0.39	4.85±0.27
	48h	57.45±2.30	17.28±1.47	2.66±0.24
	72h	88.53±0.67	79.53±0.73	49.23±5.45
Ben-Men-1	24h	39.00±2.74	8.94±0.93	4.54±0.17
	48h	59.70±1.60	15.20±0.77	3.61±0.33
	72h	85.03±1.33	76.85±0.46	46.83±1.93

Figure S1

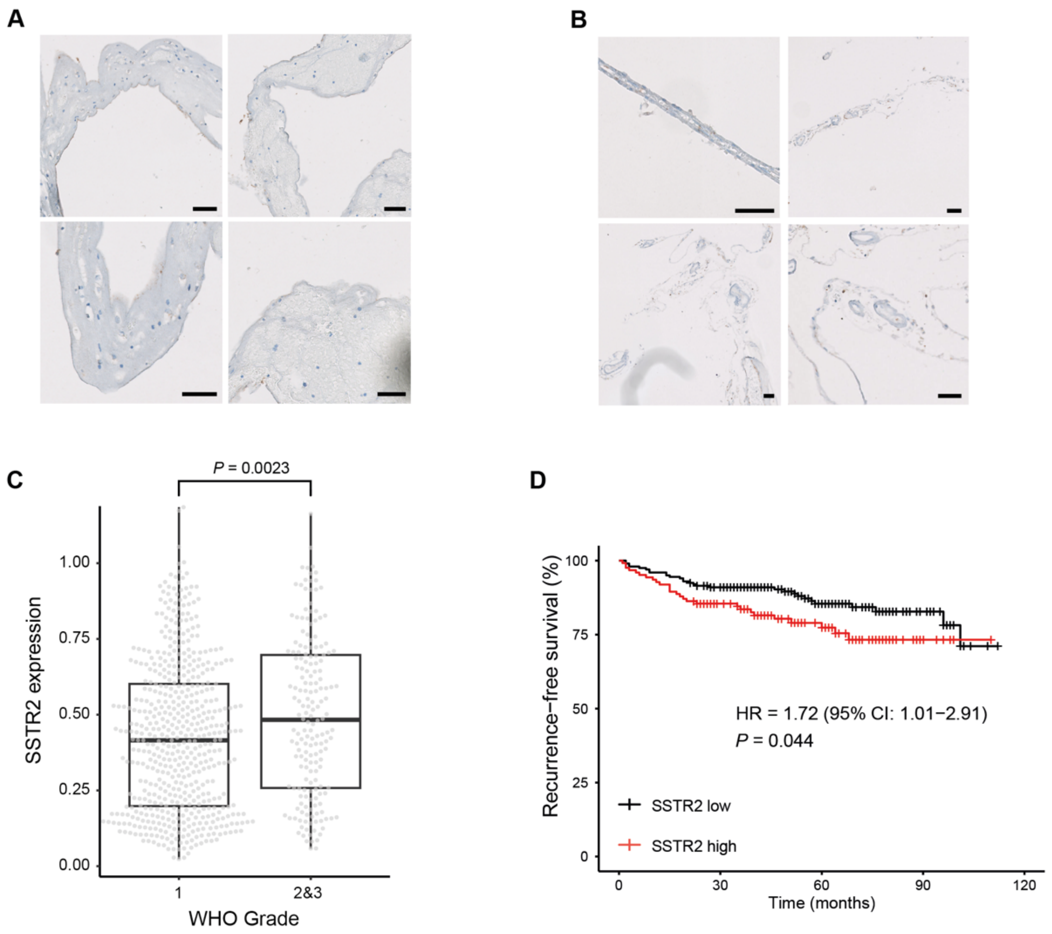

Figure S1. SSTR2 expression in human tissues. (A-B) Representative images of SSTR2 immunohistochemistry (IHC) staining on dura (A) and leptomeninges (B) of autopsy patients (N=4) without meningeal disease. Scale bar, 50 µm. (C) SSTR2 optical sum mean by World Health Organization (WHO) grade using QuPath (Wilcoxon test). (D) Recurrence-free survival of meningioma patients by SSTR2 expression determined in (C), (log-rank test). HR, hazard ratio; SSTR2 low/high, SSTR2 expression cut-off: 0.5.

Related to Figure 1.

Figure S2

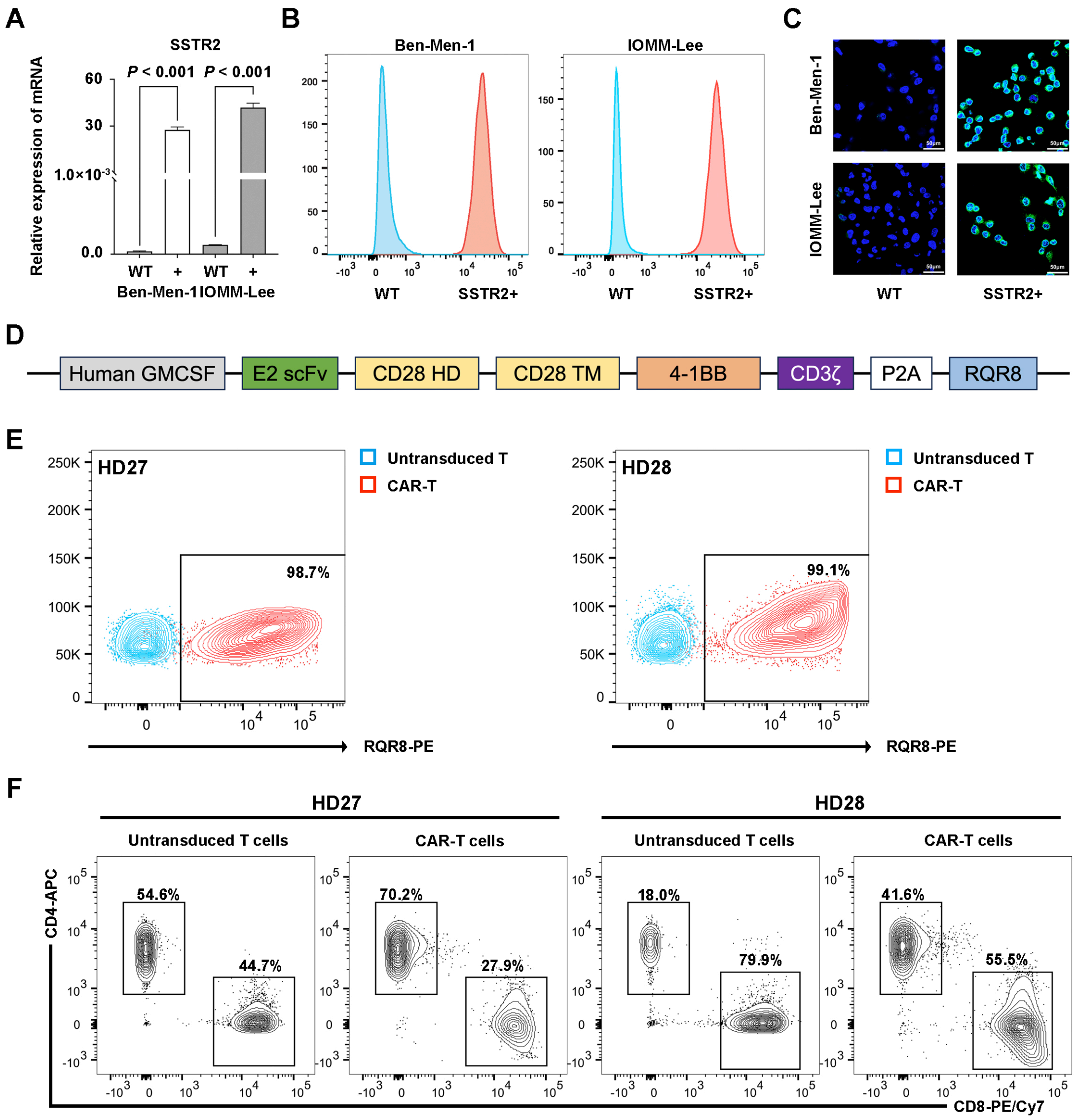

Figure S2. Generation of human SSTR2+ cell lines and AdFITC CAR-T cells. (A) SSTR2 relative mRNA expression levels of wild type (WT) and SSTR2 positive (+) Ben-Men-1 and IOMM-Lee cells were quantified by qPCR. (B) SSTR2 protein levels of WT and SSTR2+ Ben-Men-1 and IOMM-Lee cells were analyzed by flow cytometry. (C) WT and SSTR2+ Ben-Men-1and IOMM-Lee cells were incubated with octofluo at the indicated concentration for 1 h at 37 ℃ with PBS and analyzed using confocal microscopy. Hoechst (blue), octofluo (green). Scale bar, 50 µm. (D) Schematic representation of the AdFITC CAR constructs inserted into the lentivirus vector plasmid. (E) The transduction rates of AdFITC CAR-T cells analyzed by flow cytometry on day 6 were 98.7% (healthy donor [HD]27) and 99.1% (HD28), with untransduced T cells of each HD used as a control. (F) Upon transduction, the CD4:CD8 ratio of untransduced T cells and AdFITC CAR-T cells from HD27 and HD28 were

analyzed by flow cytometry. There was an expected increase in the CD4/CD8 ratio favoring CD4+ T cells in both healthy donor samples (CD4/CD8 untransduced → CAR: HD27 55/45% → 70/28%, HD28 18/80% → 42/56%).

Related to Figure 2.

Figure S3

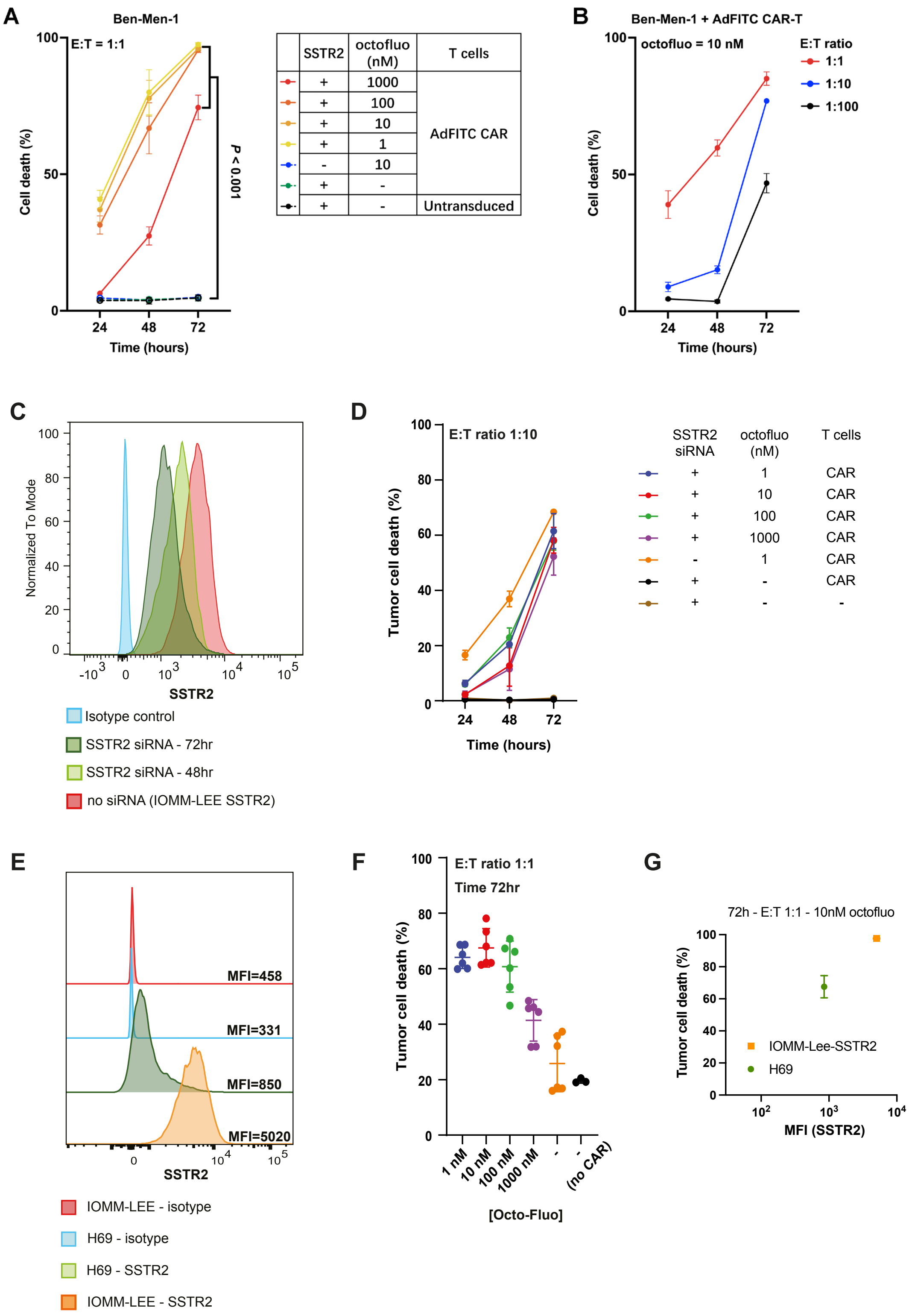

Figure S3. AdFITC(E2)-CAR T-cells cytotoxicity against Ben-Men-1 and low SSTR2-expressing cell lines. (A) Ben-Men-1 cells were incubated with AdFITC CAR-T or untransduced T cells (E:T = 1:1) and octofluo at different concentrations for 24 h, 48 h and 72 h. (B) SSTR2+ Ben-Men-1 cells were incubated with AdFITC CAR-T or untransduced T cells

at different E:T ratio and octofluo (10 nMol) for 24 h, 48 h and 72 h. (**C**) Representative flow cytometry plots of SSTR2 expression after SSTR2 siRNA transfection of the IOMM-LEE SSTR2 cells. (**D**) Co-Culture setup as in (A) against IOMM-LEE cells transfected with SSTR2 siRNA, and octofluo at different concentrations for 24 h, 48 h and 72h (n=2 donors, duplicates in one experiment). (**E**) Representative flow cytometry plots of SSTR2 expression of H69 and IOMM-LEE SSTR2 cells. (**F**) Co-Culture setup as in (A) against H69 cells and octofluo at different concentrations after 72 h. (G) Co-Culture results of Fig. 2A and Fig. S3F depending on MFI of target cell line. *Related to Figure 2.*

Figure S4

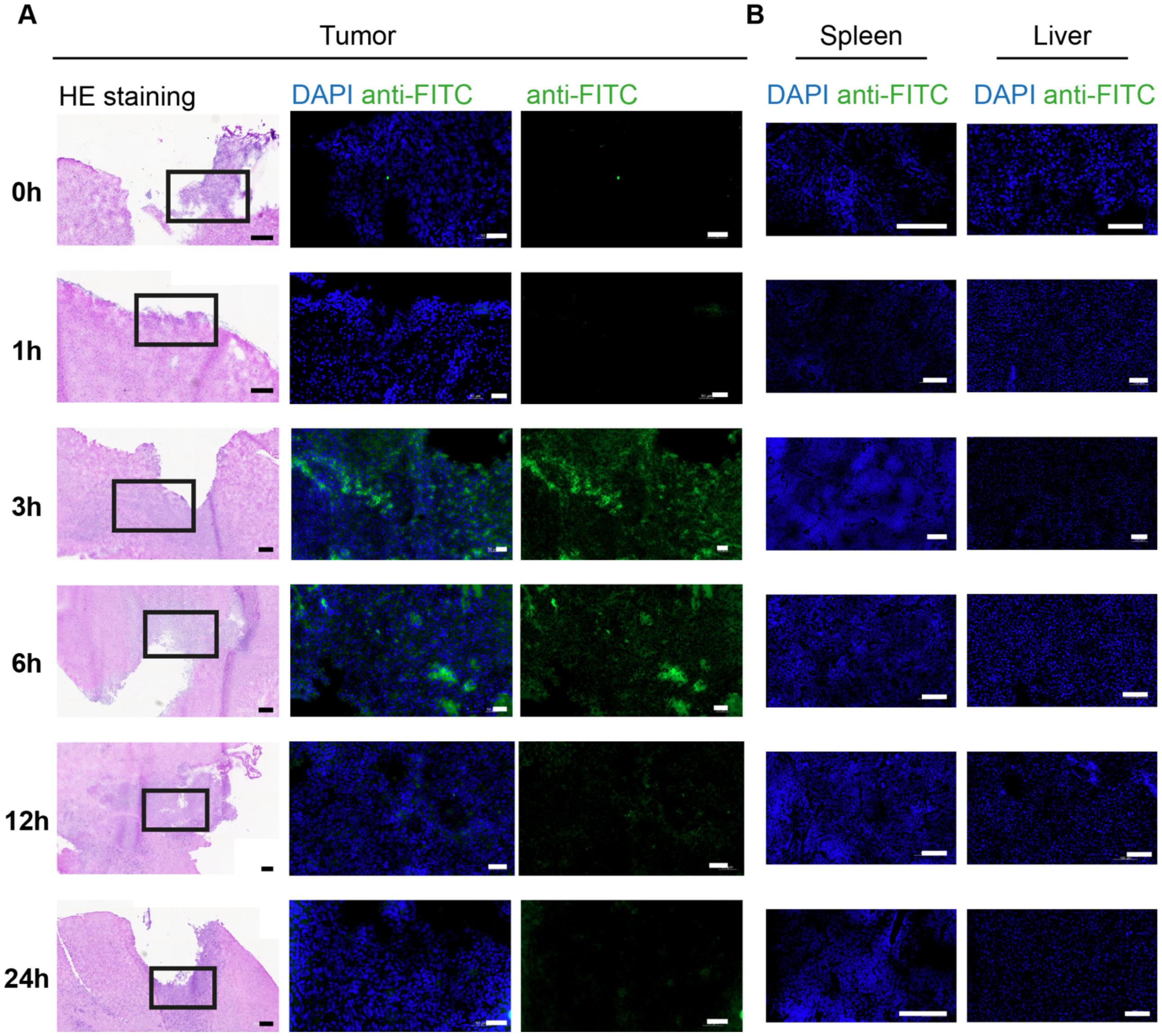

Figure S4. Biodistribution of octofluo in orthotopic IOMM-Lee meningiomas and peripheral organs. (A) SSTR2+ IOMM-Lee meningioma-bearing NOD-SCID mice were treated with 1 nMol octofluo intravenously and sacrificed at indicated time-points (0 h, 1 h, 3 h, 6 h, 12 h and 24 h). Tumor area was confirmed by HE staining and consecutive sections were stained by immunofluorescence against FITC. Black bar, 200 µm. White bar, 50 µm. (B) Spleens and livers of the same mice as in (A) were harvested and stained against FITC. White bar, 100 µm.

Related to Figure 3.

Figure S5

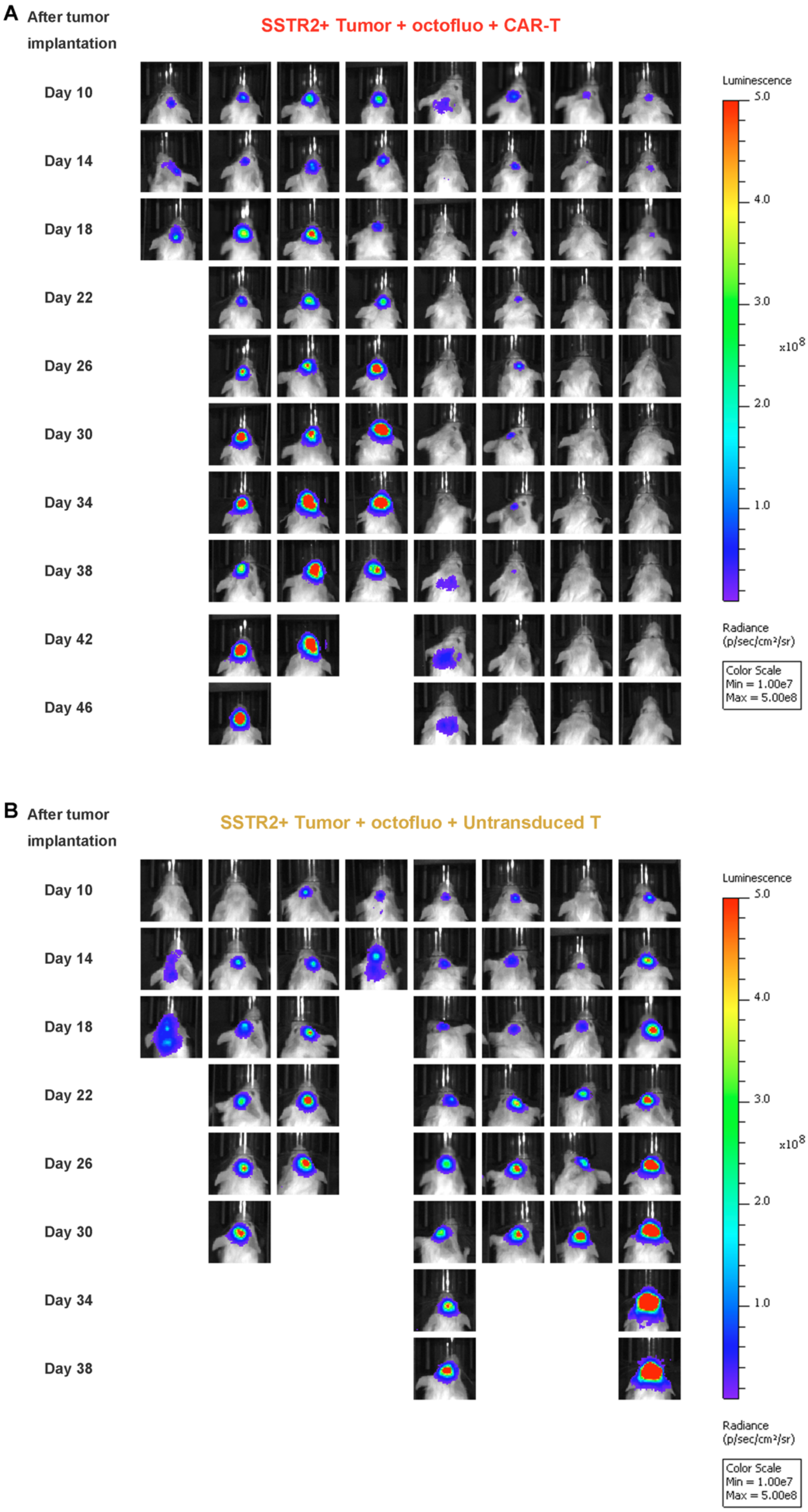

(Figure S5. Continued on next page)

Figure S5 continued

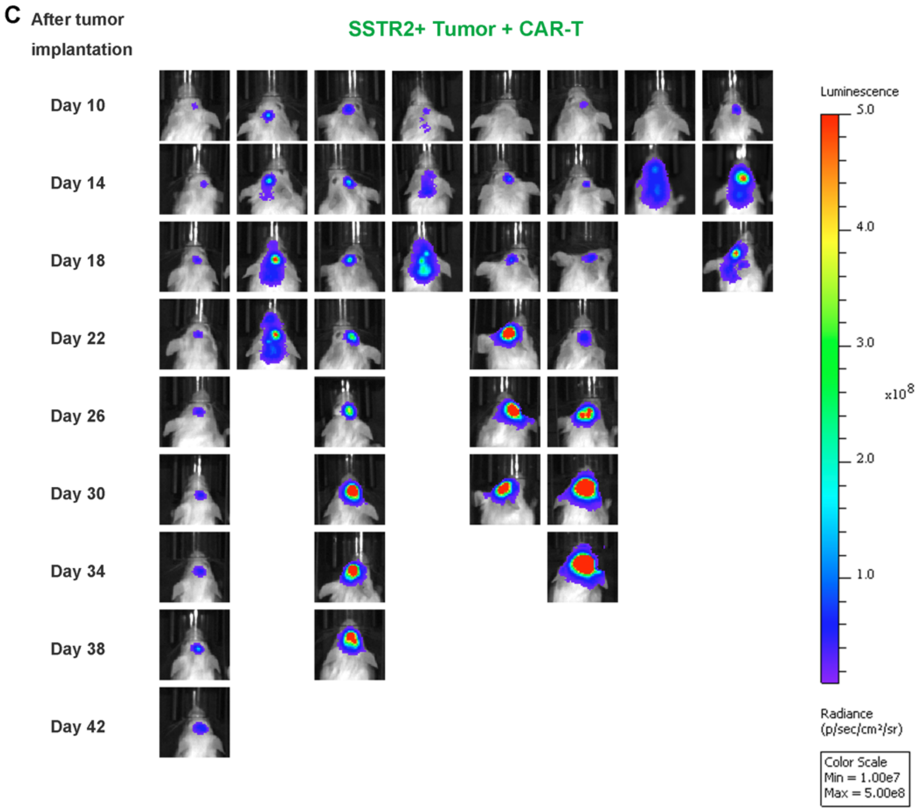

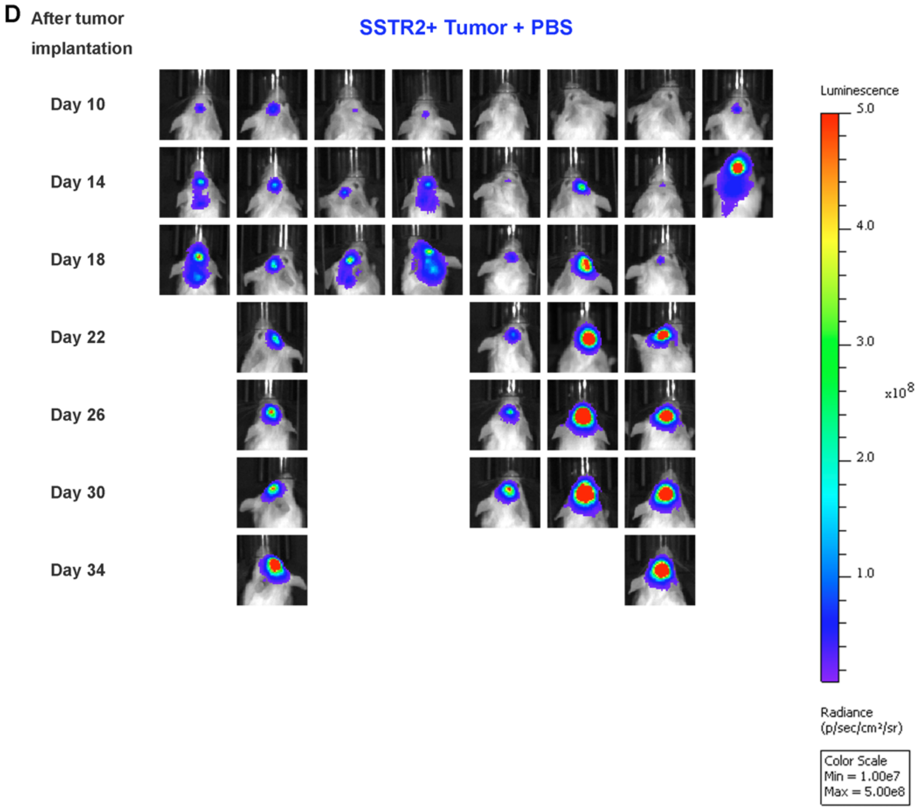

(Figure S5. Continued on next page)

Figure S5 continued

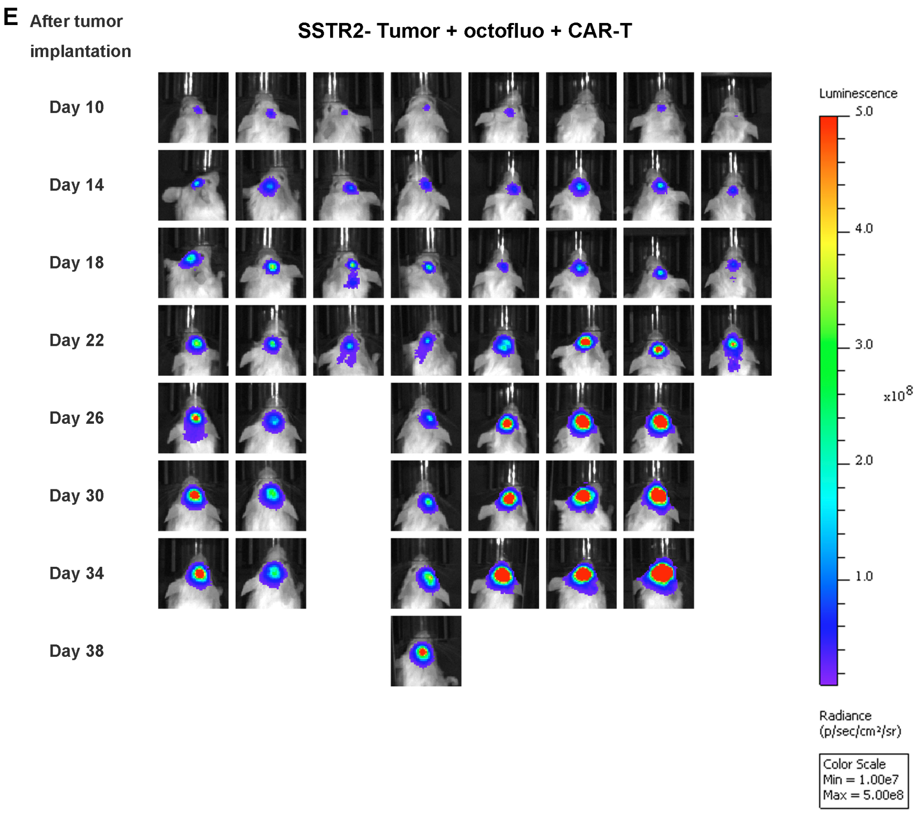

Figure S5. Bioluminescence imaging of orthotopic IOMM-Lee meningiomas. NOD-SCID mice were treated as indicated in Figure 3A. (A) AdFITC CAR-T cells i.t. with octofluo i.v. in SSTR2+ tumors; (B) Untransduced T cells i.t. with octofluo i.v. in SSTR2+ tumors; (C) AdFITC CAR-T cells i.t. in SSTR2+ tumors; (D) PBS i.v. in SSTR2+ tumors; (E) AdFITC CAR-T cells i.t. with octofluo i.v. in SSTR2- tumors. N = 8 mice per group.

Related to Figure 3.

Figure S6

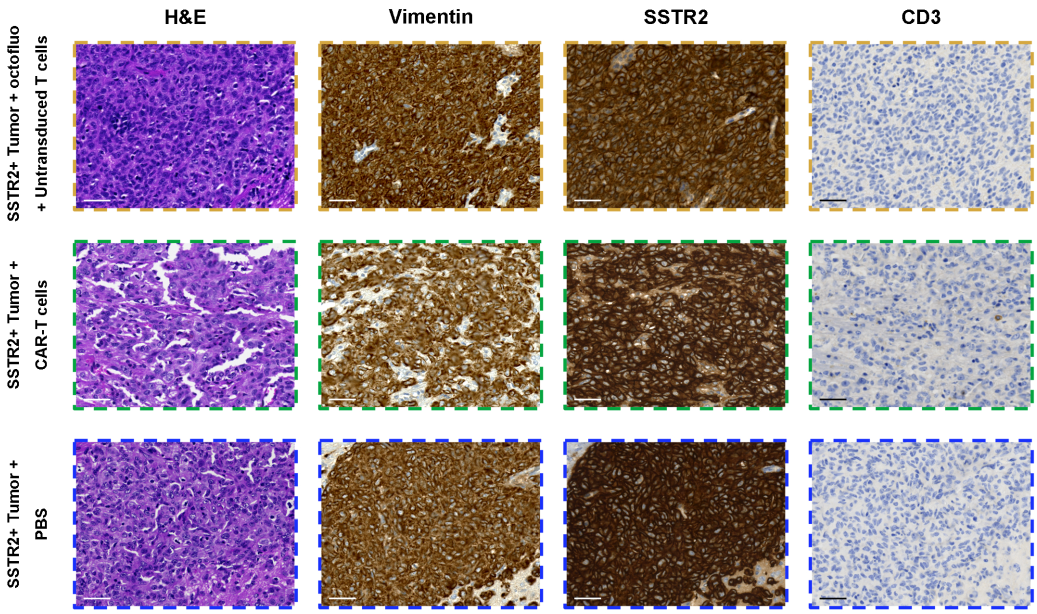

Figure S6. Histopathologic workup of orthotopic IOMM-Lee meningiomas. SSTR2+ IOMM-Lee meningioma-bearing mice were treated with different conditions. The tumor-bearing brains were isolated 1 week after first CAR-T injection and stained by H&E and immunohistochemistry (IHC) for Vimentin, SSTR2 and CD3. Representative images of H&E staining and IHC staining of Vimentin, SSTR2 and CD3 from different groups are shown. Scale bars, 100 μm.

Related to Figure 3.

Figure S7

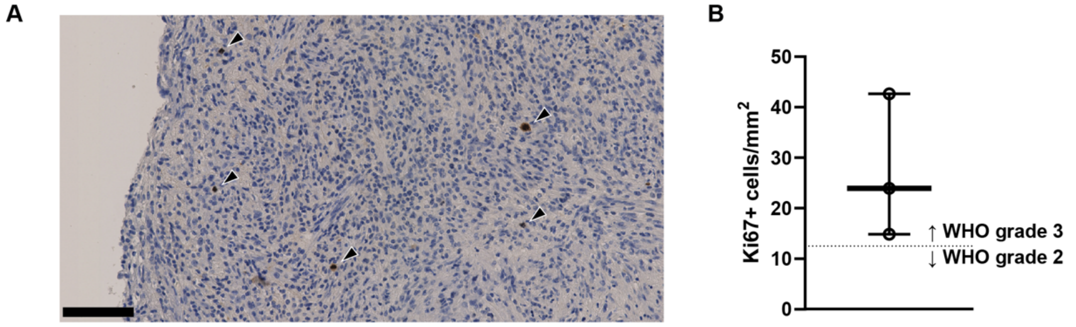

Figure S7. Mitotic index of orthotopic RCAS-induced meningiomas. (A) Representative Ki67 staining of genetically engineered murine meningiomas. Arrows indicate Ki67 positive nuclei; Scale bar, 100 µm. (B) Quantification of Ki67 positive cells per area of three RCAS-induced meningiomas. The total analyzed area was 15.05 mm^2.

Related to Figure 4.

Figure S8

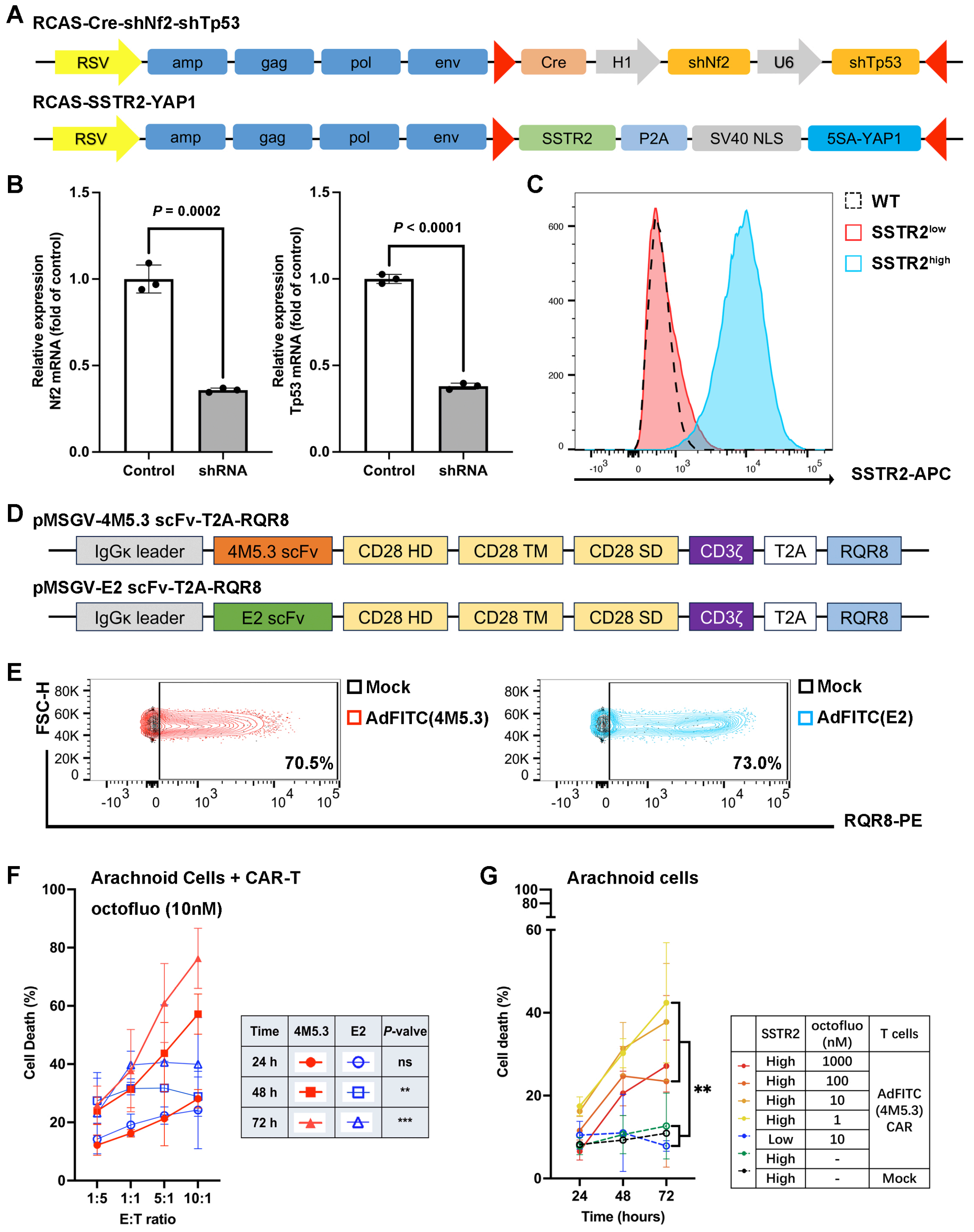

Figure S8. Genetic engineering of murine meningiomas and AdFITC CAR-T cells. (A) Schematic representation of the RCAS constructs. RCAS-Cre-shNF2-shTp53 and RCAS-SSTR2-YAP1 plasmids were used for RCAS-meningioma establishment. (B) The knockdown efficiency of shNf2 (left) and shTp53 (right) in NIH-3T3 cells was validated by qPCR. (C) The transduction of SSTR2 was validated by flow cytometry and the clones of arachnoid cells with SSTR2 low ($SSTR2^{low}$) or high ($SSTR2^{high}$) expression were sorted out by flow cytometry. (D) Schematic representation of the

AdFITC(4M5.3) and AdFITC(E2) CAR constructs inserted into the retrovirus vector plasmids. Herein, 4M5.3 had higher affinity (K_D = 300 fM) than E2 (K_D = 8.9 nM) (E) With mock transduced T cells serving as control, the transduction rate reached 70.5% for AdFITC(4M5.3) CAR-T cells and 73.0% for AdFITC(E2) CAR-T cells. (F) $SSTR2^{high}$ arachnoid cells were incubated with AdFITC(E2) CAR-T cells or AdFITC(4M5.3) CAR-T cells at different E:T ratio and octofluo (10 nMol) for 24, 48 and 72 h (Mean, 95% CI, N = 3). The killing efficiency of AdFITC(4M5.3) was around twice higher than that of AdFITC(E2) CAR-T cells. Therefore, murine AdFITC(4M5.3) CAR-T cells were used in the following *in vitro* and *in vivo* experiment for the RCAS model. (G) $SSTR2^{low}$ or $SSTR2^{high}$ arachnoid cells were incubated with AdFITC(4M5.3) CAR-T or mock T cells (E:T = 1:1) and octofluo at different concentrations for 24 h, 48 h and 72 h (Mean, 95% CI, N = 3). With 10 nMol or 1 nMol of octofluo, approximately 15%, 30%, and 40~45% of $SSTR2^{high}$ cells were killed after 24, 48 and 72 h of co-culture, respectively, and with 1000 nMol or 100 nMol of octofluo, approximately <10%, 20%, and 25% of $SSTR2^{high}$ cells were killed after 24, 48 and 72 h of co-culture, respectively, while around 90% $SSTR2^{low}$ cells or $SSTR2^{high}$ cells co-cultured with AdFITC(4M5.3) CAR-T or mock T cells were still alive.

Related to Figure 4.

Figure S9

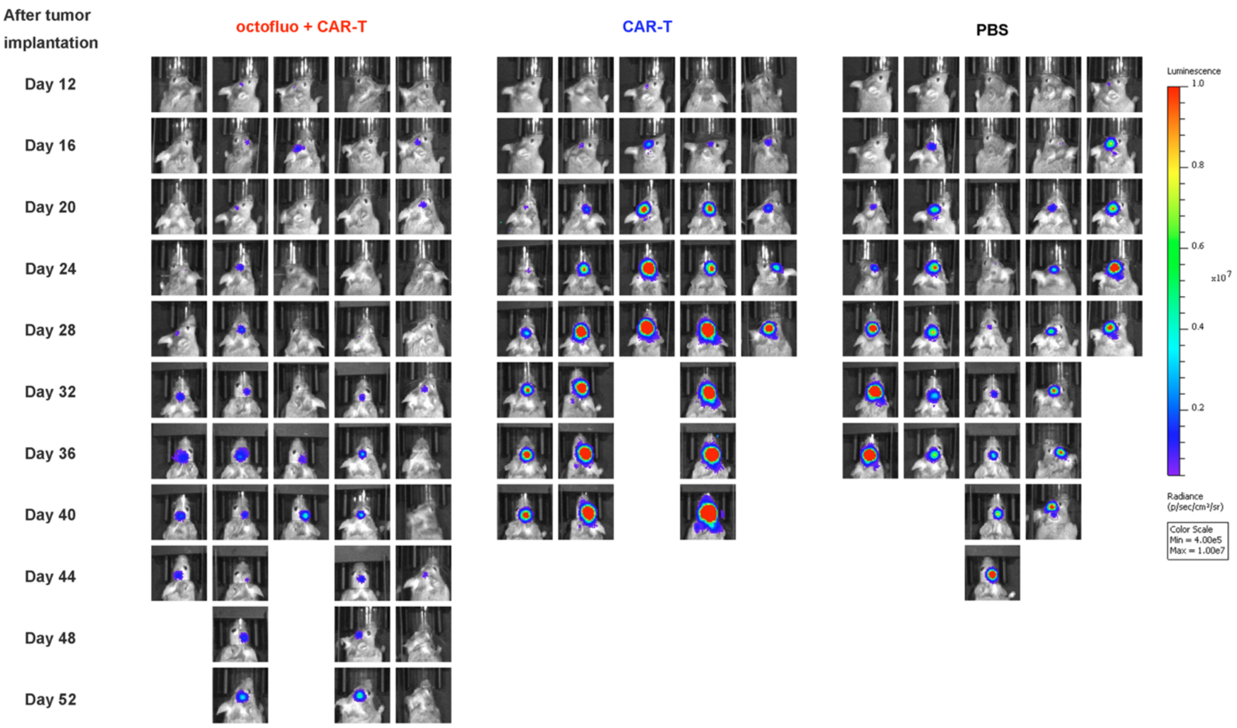

Figure S9. Bioluminescence imaging of genetically engineered meningiomas. Murine meningiomas were induced in *N/tv-a;Cdkn2a*$^{-/-}$*;Pten*$^{fl/fl}$*;Luc*$^{LSL/LSL}$ by subdural injection of DF-1 cells expressing *RCAS-SSTR2-P2A-NLS-5SA-Yap1* and *RCAS-Cre-H1-shNf2-U6-shTp53*. Mice were treated as indicated. N = 5 mice per group (of a total of N = 10 per group) were randomly selected for *in vivo* imaging. *Related to Figure 4.*

Figure S10

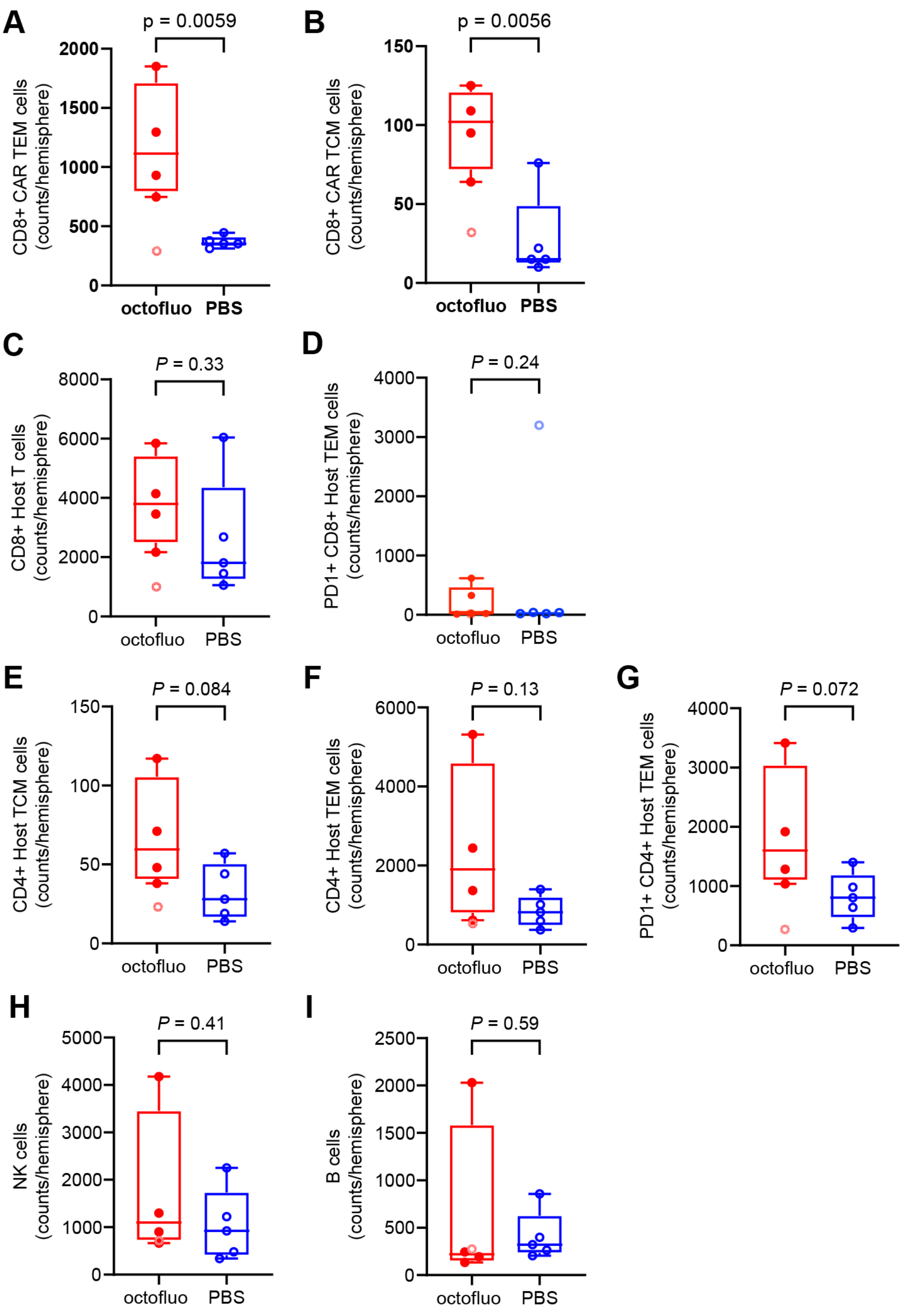

Figure S10. Host lymphocyte populations in murine meningiomas treated with AdFITC CAR-T cells. Cell populations were determined on day 7 after treatment initiation by multi-dimensional flow cytometry. (A-C) Cell counts of Cd4+ endogenous (central memory, effector memory or Pd1+ effector memory) T cell subsets (A), or of (total, effector memory or Pd1+ effector memory) Cd8+ endogenous T cells (B) or of NK cells and B cells (C) (N = 4-5/group, *t* test). One

outlier in the experimental group (open red circle) was not included in the statistical analysis for the lack of CAR-T cell expansion. One outlier in the PBS treated group was not included in the statistical analysis of D. *Related to Figure 5.*

Figure S11

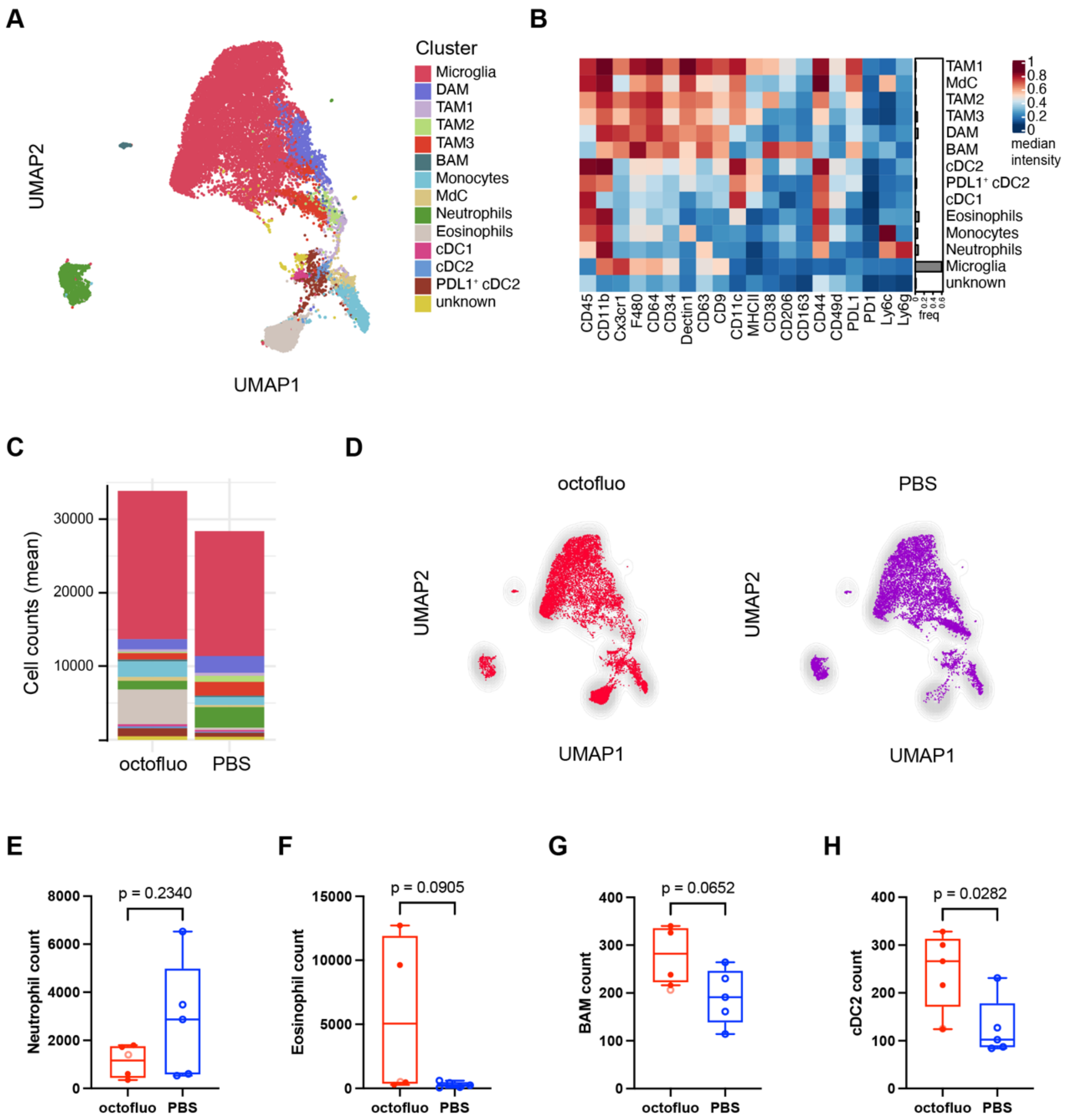

Fig. S11. Myeloid tumor microenvironment multi-dimensional flow cytometry of genetically engineered murine meningiomas treated with AdFITC CAR-T cells for 7 days.
(A) Uniform Manifold Approximation and Projection (UMAP) visualization of myeloid immune cell populations indicated in panel in meningioma bearing hemisphere (N = 5/group). (B) Heatmap of myeloid immune cell populations identified by indicated cell and phenotype marker expression. (C) Mean counts of cell populations indicated in panel (A) (n = 5/group). (D) UMAP visualization of myeloid immune cell populations depending on treatment. (E-H) Cell counts of neutrophils (E), eosinophils (F), Border associated macrophages (BAM) (G), conventional dendritic cells type 2 (cDC2) (H, N = 4-5/group, *t* test). One outlier in the experimental group (open red circle) was not included in the statistical analysis for the

lack of CAR-T cell expansion.
Related to Figure 5.

Figure S12

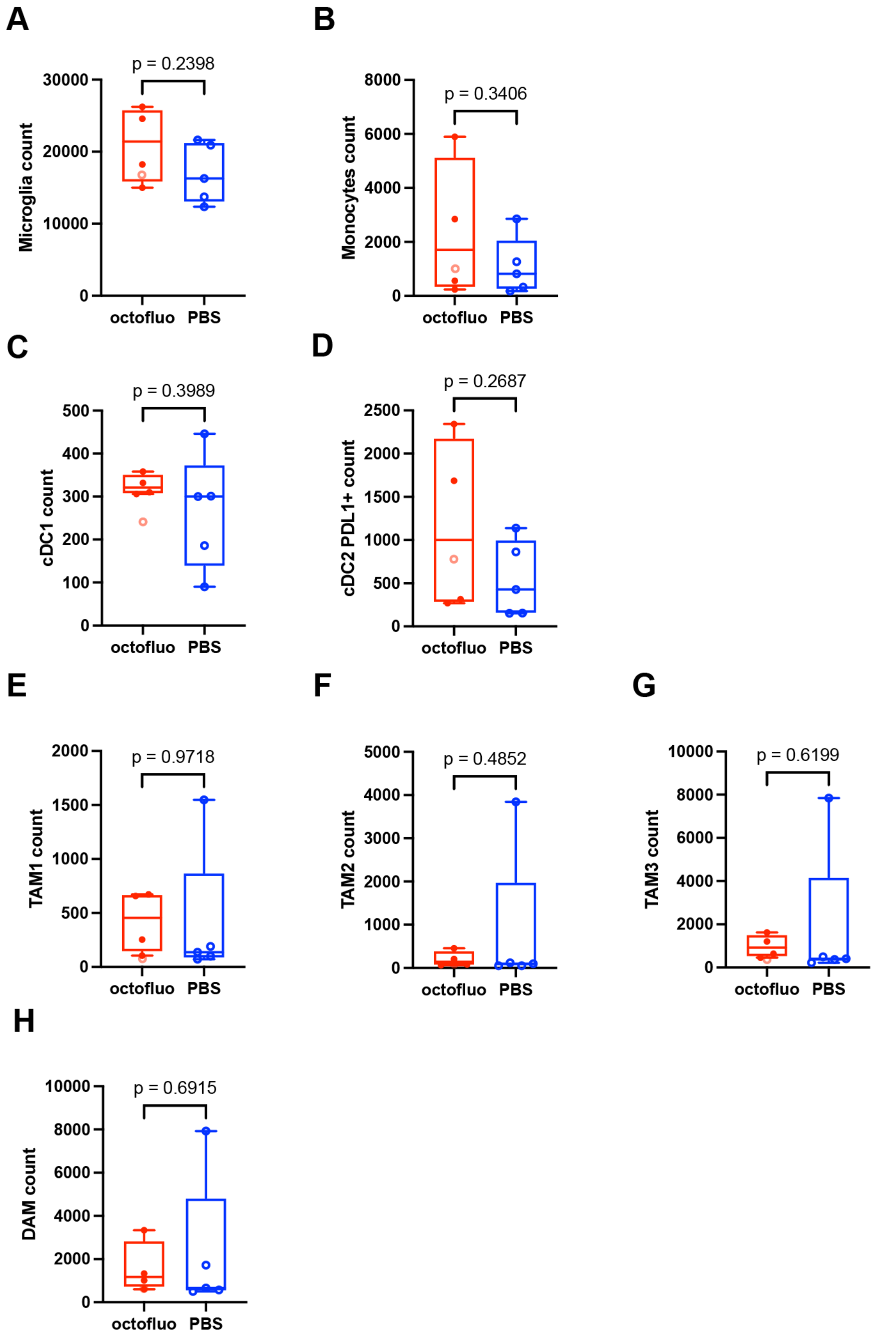

Figure S12. Host myeloid populations in murine meningiomas treated with AdFITC CAR-T cells. Cell populations were determined on day 7 after treatment initiation by multi-dimensional flow cytometry. (A, B) Cell counts of microglia and monocytes, as well as classical dendritic cells type 1 or PD-L1 expressing type 2 dendritic cells (C, D), or of tumor associated macrophages (TAM) 1-3 (E-G) or of disease associated macrophages (DAM) (H) (N = 4-5/group, *t* test). One outlier in the experimental group (open red circle) was not included in the statistical analysis for the lack of CAR-T cell expansion. *Related to Figure 5.*

References

Nassiri, F. *et al.* A clinically applicable integrative molecular classification of meningiomas. *Nature* **597**, 119-125 (2021). https://doi.org:10.1038/s41586-021-03850-3

Padevit, L. *et al.* A prognostic model for tumor recurrence and progression after meningioma surgery: preselection for further molecular work-up. *Front Oncol* **13**, 1279933 (2023). https://doi.org:10.3389/fonc.2023.1279933
